# Chromosome-scale genome assembly, carbohydrate metabolism transcriptome atlas, and candidate genes for polysaccharide accumulation in *Asparagus cochinchinensis*

**DOI:** 10.3389/fpls.2026.1867413

**Published:** 2026-06-29

**Authors:** Jun Zhao, Feili Yan, Aimeng Chen, Dan Liu, Jiahui Wu, Liuyan Wang, Siqi Liu, Youcai Chen, Wei Zhang, Ling Tang, Qi Li, Zhichao Xu, Ma Yu, Xiangyang Lyu

**Affiliations:** 1 College of Life Sciences and Agri-forestry, Southwest University of Science and Technology, Mianyang, China; 2 Neijiang Academy of Agricultural Sciences of Sichuan Province, Neijiang, China; 3 Crop Research Institute of Sichuan Academy of Agricultural Sciences, Chengdu, China; 4 Neijiang Dongxing District Traditional Chinese Medicine Great Health Industry Promotion Center, Neijiang, China; 5 College of Life Science, Northeast Forestry University, Harbin, China

**Keywords:** *Asparagus cochinchinensis* (Lour.) Merr., candidate genes, chromosome-level genome, evolution, genome assembly, polysaccharide biosynthesis

## Abstract

*Asparagus cochinchinensis* is a valued medicinal plant, whose bioactive polysaccharides are important secondary metabolites. However, the evolutionary origins and spatiotemporal regulation of their biosynthesis remain unclear, hindered by the lack of a reference genome. Here, by combining PacBio HiFi, Illumina, and Hi-C sequencing technologies, we generated the first publicly available chromosome-level genome assembly of *A. cochinchinensis* (1.52 Gb; 10 pseudochromosomes). We annotated 48, 453 protein-coding genes and dated the divergence of *Asparagus* from Orchidaceae to ~95 million years ago. Comparative genomics revealed lineage-specific whole-genome duplication events and gene family expansions linked to metabolic adaptation. To identify candidate genes associated with polysaccharide accumulation, we generated transcriptomes from root tuber, fibrous root, leaf, and spear tissues. Co-expression network and pathway analyses identified 16 key genes significantly associated with polysaccharide accumulation, particularly in underground storage organs. Our analysis reconstructs the sucrose-to-polysaccharide biosynthetic pathway and highlights differential gene expression between tuber and fibrous root tissues, revealing the spatial compartmentalization of this metabolic process. This high-quality genome provides a foundational resource for elucidating the evolution and tissue-specific regulation of secondary metabolite biosynthesis in *Asparagus*, with implications for molecular breeding aimed at enhancing medicinal compound yield.

## 1 Introduction

*Asparagus cochinchinensis* (Lour.) Merr., a species within the *Asparagus* genus of the Asparagaceae family (subfamily Asparagoideae, order Asparagales), is distributed across several East and Southeast Asian countries, including China, Japan, Korea, and Vietnam (Liu et al., 2024). It holds a significant place in traditional herbal medicine, having been first documented in the ancient Chinese pharmacopoeia *Shennong’s Classic of Materia Medica*. Its longstanding medicinal application has been substantiated by extensive clinical practice (Wang et al., 2022). The dried roots of *A. cochinchinensis* serve as its primary medicinal part. For centuries, they have been utilized in China, either independently or in combination with other herbs, for the treatment of conditions such as asthma, cough, constipation, thrombosis, and inflammatory diseases (Wang et al., 2022) The plant is incorporated into numerous classical formulations that have been widely employed in medical practice, contributing substantially to public health, particularly in China and other Asian regions (Alok et al., 2013). Beyond its therapeutic uses, *A. cochinchinensis* also finds applications in health products, functional foods, and cosmetics (Wang et al., 2022). It is consumed as a dietary or nutritional supplement (Zabotina et al., 2021), formulated into cosmetics for whitening and anti-aging purposes, and even employed as a raw material in fermentation and winemaking processes. As an important medicinal herb, *A. cochinchinensis* has garnered considerable research attention in recent decades. Significant progress has been made in isolating and characterizing its bioactive constituents; to date, nearly 100 compounds have been identified, primarily including steroidal saponins, C21-steroids, lignans, polysaccharides, and amino acids (Zhang et al., 2021). With the deepening exploration of traditional medicine, the potential of herbs such as *A. cochinchinensis* in disease prevention and treatment continues to expand. Consequently, given its considerable therapeutic potential and broad prospects for development, *A. cochinchinensis* merits further in-depth investigation.

Polysaccharides constitute the primary active constituents of *A. cochinchinensis*. *A. cochinchinensis* polysaccharides (ACPs) are composed of various monosaccharide units, including glucose, arabinose, galactose, xylose, and rhamnose, linked by glycosidic bonds, and also contain other diverse compounds (Li et al., 2024). In *A. cochinchinensis*, amino acid polysaccharides exhibit a broad molecular weight range spanning from several thousand to several million daltons. Their biological activity is determined by structural characteristics such as molecular weight, degree of branching, sugar chain composition, and types of glycosidic linkages (Lei et al., 2017). ACP extracted from the root tuber of *A. cochinchinensis* has demonstrated significant anti-multiple myeloma activity, though its antitumor effect may be attenuated under certain conditions (Zabotina et al., 2021). Similarly, other studies have reported that ACP exhibits notable activity against myelodysplastic cells and can mitigate the suppression of tumor immune responses (Li et al., 2020). These findings suggest the potential application of ACP in future cancer therapies. It is noteworthy that ACP with higher molecular weight generally has stronger immunomodulatory and antitumor activities. Previous studies have shown that ACP can inhibit the expression of HIF1α and VEGF in human hepatocellular carcinoma cells (SK-Hep1 and Hep-3B) under both normoxic and hypoxic conditions, thereby suppressing their proliferation, migration, invasion, and angiogenesis (Cheng et al., 2019). Furthermore, water-extracted components from *A. cochinchinensis*—including polysaccharides, saponins, quercetin, β-sitosterol, and 7-methoxy-2-methylisoflavone—exert synergistic effects in inhibiting colon cancer proliferation (Cheng et al., 2019; Liang et al., 2022). Owing to its potent scavenging effects on superoxide anions and hydroxyl radicals, ACP holds potential for the prevention and treatment of cardiovascular diseases, as well as for anti-aging and radioprotective applications (Almatroodi et al., 2021). Additionally, ACP exhibits a range of other bioactivities, such as inhibiting oxidative stress, reducing levels of malondialdehyde, nitric oxide, and pro-inflammatory cytokines, while enhancing the activities of reduced glutathione, superoxide dismutase (SOD), and catalase. These actions collectively contribute to reduced brain injury, positioning ACP as a promising neuroprotective agent (Li et al., 2024).

Although the chromosome-level genome of the congeneric species *A. officinalis* has been published (Harkess et al., 2017), and an *A. cochinchinensis* genome project has been registered in NCBI under PRJNA805610, no publicly available chromosome-level genome assembly or fully annotated genomic resource for *A. cochinchinensis* is currently available. This lack of a high-quality reference genome has limited the systematic identification of carbohydrate metabolism genes associated with polysaccharide accumulation in *A. cochinchinensis*. To address this gap, we generated the first publicly available chromosome-scale reference genome for *A. cochinchinensis*. We hypothesize that a chromosome-scale genome assembly, combined with comparative genomics and transcriptomics, will enable the systematic identification of key genes and the reconstruction of polysaccharide biosynthetic pathways. Accordingly, the primary objectives of this study are: (1) to generate a high-quality, chromosome-level genome assembly of *A. cochinchinensis* using an integrated sequencing approach; (2) to perform comparative genomic and phylogenetic analyses to elucidate its evolutionary history and gene family dynamics; and (3) to integrate RNA-seq data from multiple tissues to reconstruct the polysaccharide biosynthesis pathway and identify candidate genes regulating polysaccharide accumulation. The genomic resource generated here will not only serve as a foundation for future molecular studies but also facilitate breeding programs aimed at improving medicinal quality and stress resistance in *Asparagus* species.

## 2 Materials and methods

### 2.1 Plant Materials

*Asparagus cochinchinensis* (Lour.) Merr. specimens were collected from various regions across China. In total, eight distinct *Asparagus* species were included in this study (**Table 1**). Detailed sample information is as follows: From Neijiang City, Sichuan Province, China, we collected a wild-type plant, a male plant, a widely cultivated cultivar ‘Neijiang 1’, and a female plant of *A. cochinchinensis*. Another cultivar of *A. cochinchinensis*, ‘Guidong 1’, which is also extensively cultivated, was obtained from Nanning City, Guangxi Zhuang Autonomous Region, China. An additional *A. cochinchinensis* plant was sampled from Yulin City, Guangxi. Furthermore, two individuals of *Asparagus taliensis* were collected from Wuchuan County, Guizhou Province, and Yunnan Province, China, respectively. The male plant of *A. cochinchinensis* collected from Neijiang City was selected for whole-genome sequencing. For transcriptomic analysis, root tuber (RT), fibrous root (FR), leaf (LE), and spear (SP) tissues were separately collected from each of the eight species. All samples for genome and RNA sequencing were processed with three biological replicates. Immediately after collection, samples were flash-frozen in liquid nitrogen and stored at –80°C. For each *A. cochinchinensis* sample, three plants were randomly selected as replicates for morphological characterization of root tubers. Measured traits included number, length, diameter, weight, shape, and polysaccharide content.

**Table 1:** Plant materials used in this study.

Number	Species	Producing area	Tissues			
1~12	*Asparagus cochinchinensis*(Lour.)Merr (Wild type)	Neijiang, Sichuan	RT (1,2,3)	FR (4,5,6)	LE (7,8,9)	SP (10,11,12)
13~24	*Asparagus cochinchinensis*(Lour.)Merr (Male plant)	Neijiang, Sichuan	RT (13,14,15)	FR (16,17,18)	LE (19,20,21)	SP (22,23,24)
25~36	*Asparagus cochinchinensis*(Lour.)Merr (Neijiang 1)	Neijiang, Sichuan	RT (25,26,27)	FR (28,29,30)	LE (31,32,33)	SP (34,35,36)
37~48	*Asparagus cochinchinensis*(Lour.)Merr (Female plant)	Neijiang, Sichuan	RT (37,38,39)	FR (40,41,42)	LE (43,44,45)	SP (46,47,48)
49~60	*Asparagus cochinchinensis*(Lour.)Merr (Guidong 1)	Nanning, Guangxi	RT (49,50,51)	FR (52,53,54)	LE (55,56,57)	SP (58,59,60)
61~72	*Asparagus cochinchinensis*(Lour.)Merr	Yulin, Guangxi	RT (61,62,63)	FR (64,65,66)	LE (67,68,69)	SP (70,71,72)
73~84	*Asparagus taliensis*	Wuchuan, Guizhou	RT (73,74,75)	FR (76,77,78)	LE (79,80,81)	SP (82,83,84)
85~96	*Asparagus taliensis*	Kunming, Yunnan	RT (85,86,87)	FR (88,89,90)	LE (91,92,93)	SP (94,95,96)

RT, Root Tuber; FR, Fibrous Root; LE, Leaf; SP, Spear. Ac1, 1~12; Ac2, 13~24; Ac3, 25~36; Ac4, 37~48; Ac5, 49~60; Ac6, 61~72; Ac7, 73~84; Ac8, 85~96.

### 2.2 Genome sequencing and assembly

Genomic DNA was extracted from fresh tender tissues of a male *A. cochinchinensis* plant collected from Neijiang using the TIANamp Plant DNA Kit (Tiangen, Beijing, China) following the manufacturer’s instructions. The extracted DNA was sheared using a sonication device, and short-insert (150 bp) paired-end (PE) sequencing libraries were prepared from the fragmented DNA using the Illumina Nextera DNA Library Prep Kit (Illumina, United States). All constructed libraries were sequenced on an Illumina NovaSeq 6000 platform (San Diego, CA, United States). For PacBio library construction, genomic DNA was sheared to approximately 20 kb fragments, and fragments smaller than 7 kb were filtered out using BluePippin (Sage Science, MA, United States). The filtered DNA was then converted into SMRTbell libraries using the PacBio DNA Template Preparation Kit (Pacbio, United States) according to the manufacturer’s protocol. Single Molecule Real-Time (SMRT) sequencing was performed on a PacBio Sequel II platform using HiFi Bundle (v2) sequencing reagents and 8M SMRT Cells. For Hi-C library preparation, leaves of *A. cochinchinensis* fixed in 1% (v/v) formaldehyde were used. Nucleus extraction, permeabilization, chromatin digestion, and proximity ligation were carried out with MboI (NEB, United States) as the restriction enzyme, following a previously described protocol (Xie et al., 2015). The resulting Hi-C libraries were sequenced on an Illumina NovaSeq 6000 platform with 2 × 150 bp reads. To support protein-coding gene prediction, RNA was extracted from root tuber (RT), fibrous root (FR), leaf (LE), and spear (SP) tissues using the TRNzol Universal Reagent (TIANGEN Biotech, China). RNA-Seq libraries were constructed with the NEBNext Ultra RNA Library Prep Kit (Illumina, United States) according to the manufacturer’s instructions and subsequently sequenced on an Illumina NovaSeq 6000 platform (San Diego, CA, United States). Raw reads from all sequencing platforms were subjected to quality control using Trimmomaticv0.39 (Bolger et al., 2014).

### 2.3 Genome size and heterozygosity estimation

To determine the genome size and heterozygosity of *A. cochinchinensis*, we performed a k-mer frequency analysis (Liu et al., 2020) using high-quality Illumina short reads of Q20 or above. The k-mer spectrum was generated with Jellyfish (v1.1.11) (Marçais and Kingsford, 2011) based on 17-mers. Genome size was initially calculated as the total k-mer count divided by the mean k-mer depth. For a more refined assessment, GenomeScope (v1.0.0) (Vurture et al., 2017) was subsequently employed to model the distribution and provide estimates of both genome size and heterozygosity rate.

### 2.4 Genome assembly and quality control

The chromosome-level genome was assembled through a multi-step process. First, primary contigs were constructed by integrating PacBio HiFi reads with paired-end Hi-C reads using HiFiasm v0.14 (parameters: -t 32, -f 39) (Cheng et al., 2021). To enhance base-level accuracy, Illumina short reads were aligned to these contigs with BWA-MEM (v0.7.17) (Li and Durbin, 2009), followed by iterative polishing using Pilon (v1.2) (Walker et al., 2014). Subsequently, Hi-C data were processed with the HiC-Pro pipeline and default parameters (LIGATION_SITE = GATC) (Servant et al., 2015) to generate valid interaction pairs. Contig ordering, orientation, and scaffolding into pseudochromosomes were performed with the 3DDNA pipeline, with manual curation and visualization of contact maps in Juicebox v2.20 (Durand et al., 2016). Finally, assembly completeness was assessed using BUSCO v5 (Simão et al., 2015) against a core set of conserved eukaryotic genes.

### 2.5 Genome annotation

To annotate the genome, we first constructed a *de novo* repeat library using RepeatModeler v2.0.6 (Flynn et al., 2020). This library served as a basis for RepeatMasker v4.1 (Tarailo-Graovac and Chen, 2009) to identify and mask repetitive sequences. In addition, transposable elements (TEs) were detected at both DNA and protein levels through alignment of the genome against the Repbase TE database (Bao et al., 2015). For protein-coding gene prediction, we integrated three complementary approaches: *ab initio*, homology-based, and transcriptome-supported prediction. The *ab initio* predictions were performed with Augustus (v2.5.5) (Stanke et al., 2008)and GeneMark (v4.32) (Besemer et al., 2001). Homology-based gene models were generated by aligning the genome to related species using Exonerate (v2.2.0) (Slater and Birney, 2005). Transcriptomic evidence was incorporated by mapping RNA-Seq reads to the genome assembly with TopHat (v2.1.0) (Trapnell et al., 2009), followed by transcript assembly and gene model inference with Cufflinks (Trapnell et al., 2012). All predicted gene structures were consolidated into a non-redundant set with EvidenceModeler (EVM) (Haas et al., 2008). Functional annotations were then assigned by conducting BLASTP (Camacho et al., 2009) searches (E-value ≤ 1×10^-5^) against public databases, including eggNOG (Huerta-Cepas et al., 2019), Pfam (Finn et al., 2011), GO (Ashburner et al., 2000), COG (Tatusov et al., 2000), EC (Bairoch, 2000), and KEGG (Kanehisa and Goto, 2000). Protein domains were identified via HMMER against the Pfam database, while KEGG and GO (Gene Ontology) terms were assigned to elucidate potential metabolic pathways and gene functions.

### 2.6 Phylogenetic analysis and divergence time estimation

We performed phylogenetic analysis by constructing phylogenetic trees based on single-copy genes from orthogroups across eight species. Clustering analyses were carried out using the Markov clustering algorithm implemented in OrthoFinder (v2.5.3) applied to protein sequences (Emms and Kelly, 2015). With the NCBI-NR database (Pruitt et al., 2007) as a reference, all-versus-all BLASTP searches were conducted on the peptide sequences, applying a threshold of E-value ≤ 1×10^-5^. The resulting sequences were subsequently clustered via the MCL (Enright et al., 2002) algorithm with an inflation parameter set to 1.5. Orthologous alignments were generated with MUSCLE (v3.6) (Edgar, 2004), and a custom Perl script was employed to concatenate these alignments into a single multiple sequence alignment. A neighbor-joining phylogenetic tree was reconstructed using MEGA5 software (v5.2) (Tamura et al., 2011). Molecular clock calibration and estimation of divergence times among species were performed jointly with r8s (Sanderson, 2003), with a strict molecular clock model. The calibration node was constrained using the divergence time between *A. thaliana* and *O. sativa* (142.1–163.5 Mya) as lower and upper bounds. Maximum likelihood phylogeny and corresponding branch lengths were computed with RAxML (v8.2.10) (Stamatakis, 2014) under 1, 000 bootstrap replicates. Finally, a fossil-calibrated timescale and the evolutionary history of these species were obtained using TIMETREE (Kumar et al., 2017).

### 2.7 Expansion and contraction of the gene families

A systematic comparison based on different genes was conducted to clarify the evolutionary relationship between *A. cochinchinensis* and other plant species. To better understand the evolutionary dynamics of gene gain and loss—primary drivers of functional divergence across species—we analyzed whole protein-coding gene sets from the *A. cochinchinensis* genome and seven other species, examining expansions and contractions of orthologous gene clusters among these eight lineages. Using CAFE software (v4.0) (De Bie et al., 2006), we performed computational analyses of gene family evolution. A global birth–death rate (λ) and a global error model were estimated from the dataset using maximum likelihood. Gene families exhibiting significant expansions or contractions were identified based on a P-value threshold of 0.01. Changes in gene family size were determined by comparing cluster sizes between each extant species and their respective ancestors. Gene gain and loss events along each branch of the RAxML tree were inferred with CAFE’s stochastic birth–death model. Subsequently, expanded and contracted gene families (relative to ancestral states) identified across the studied species were compared with those in *A. cochinchinensis*, providing insight into the evolutionary trajectory of gene families in this species.

### 2.8 Identification and analysis of genes that involved in the biosynthesis of *A. cochinchinensis* polysaccharides

To identify genes involved in the biosynthesis of *A. cochinchinensis* polysaccharides, root tuber (RT), fibrous root (FR), leaf (LE), and stem (SP) tissues were separately collected from each of the eight species for transcriptomic analysis. Weighted Gene Co-expression Network Analysis (WGCNA) (Langfelder and Horvath, 2008) was performed to reveal module–trait relationships between co-expression modules and key phenotypic traits of *A. cochinchinensis*, with up-regulated differentially expressed genes (DEGs) in the root tuber also being selected for further study. For polysaccharide content—the main focus of this study—a Venn analysis was conducted using genes present in both the WGCNA results and the set of root-tuber up-regulated DEGs. Functional enrichment analyses (GO and KEGG) were then performed on these overlapping genes to identify those associated with polysaccharide biosynthesis and accumulation. Additionally, to pinpoint key genes specifically involved in polysaccharide production, we selected DEGs that were up-regulated exclusively in the root tuber of accession Ac5, which exhibits the highest polysaccharide content. GO and KEGG enrichment analyses were carried out on this Ac5-specific gene set. Based on polysaccharide biosynthesis-related genes that showed high expression in root tuber and fibrous root tissues, a differential expression pathway diagram for polysaccharide biosynthesis in *A. cochinchinensis* was constructed. Finally, by integrating the results from the above analyses, several candidate key genes implicated in *A. cochinchinensis* polysaccharide biosynthesis and accumulation were identified.

## 3 Results

### 3.1 Genome size estimation and assembly of *A. cochinchinensis*

**Figure 1:**
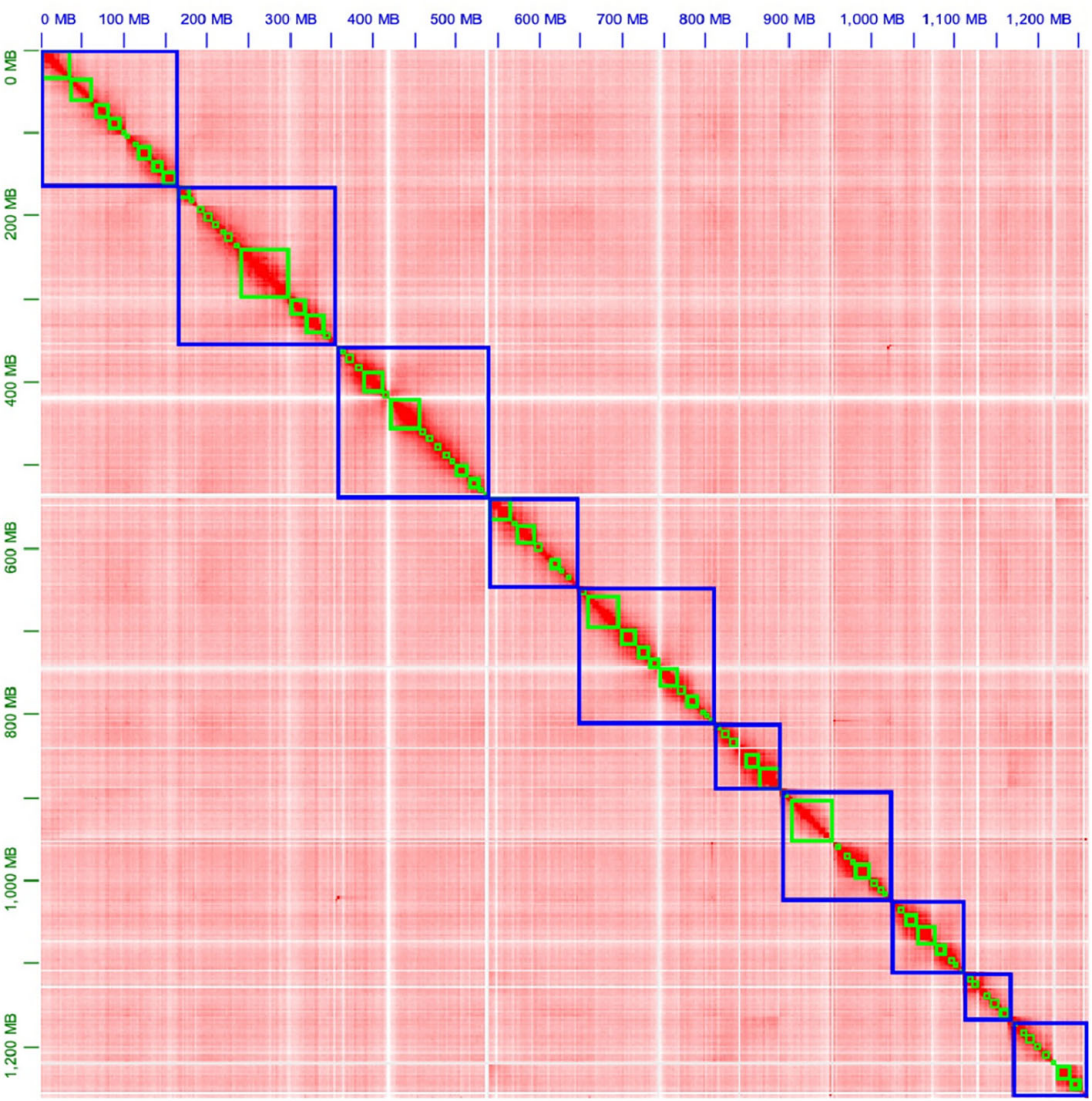
The Hi-C contact map of the *A. cochinchinensis* genome. The map illustrates the three-dimensional organization and physical interactions between different genomic regions. The intensity of contact signals, represented by the color gradient from white (low) to red (high), reflects the spatial proximity and interaction frequency along the chromosomes. Strong interactions (red) typically indicate regions that are close in the 3D nuclear space, which often correspond to topological domains or functional chromatin loops.

Prior to genome assembly, we estimated genome size, heterozygosity rate, and repetitive sequence content through K-mer analysis of Illumina clean reads. Our analysis indicated that the *A. cochinchinensis* genome is approximately 1.52 Gb in size, with a heterozygosity level of 0.725% (**Supplementary Figure 1**). A final assembly of 1.52 Gb was obtained, comprising 145 scaffolds, after polishing primary contigs with clean Illumina reads; the scaffold N50 was 157.6 Mb. Using Hi-C data, scaffolds were further assembled into pseudochromosomes, with 95.29% successfully anchored to 10 pseudochromosomes ranging in size from 55.55 to 188.24 Mb (**Table 2**). The overall GC content of the assembly was 39.86% (**Tables 3**, **4**; **Figure 1**).

**Table 2:** Summary of the ten pseudochromosomes in the *A. cochinchinensis* genome assembly.

Chromosome number	Length (bp)
chr01	166,977,632
chr02	192,760,366
chr03	180,626,159
chr04	107,351,973
chr05	165,252,557
chr06	80,610,128
chr07	132,525,470
chr08	87,053,506
chr09	56,879,882
chr10	92,281,798
unanchored	62,396,926

**Table 3:** Statistics of genome assembly of *A. cochinchinensis* at chromosome level.

Item	The chromosome-level genome assembly
Total sequences	145
Unmapped sequence	135
Total bases	1,324,716,397
Min sequence length (bp)	1,000
Max sequence length (bp)	192,760,366
Average sequence length (bp)	9,135,974.15
Median sequence length (bp)	96,547.00
N25 length (bp)	180,626,159
N50 length (bp)	165,252,557
N75 length (bp)	92,281,798
N90 length (bp)	80,610,128
N95 length (bp)	56,879,882
As	30.08%
Ts	30.06%
Gs	19.93%
Cs	19.93%
(A + T)s	60.14%
(G + C)s	39.86%

**Table 4:** Summary statistics for the repeat elements found in the *A.cochinchinensis* genome assembly.

Item	number	length occupied (bp)	percentage of sequence
sequences	145		
total length		1,324,716,397	
GC level			39.86%
bases masked:		1,043,526,576	78.77%
Retroelements	512,028	627,530,433	47.37%
SINEs	9,436	2,512,507	0.19%
Penelope	2,285	384,767	0.03%
LINEs	48,944	46,499,266	3.51%
CRE/SLACS	67	37,449	0.00%
RTE/Bov-B	35,604	36,830,061	2.78%
L1/CIN4	9,502	7,978,653	0.60%
LTR elements	453,648	578,518,660	43.67%
BEL/Pao	899	399,295	0.03%
Ty1/Copia	152,159	153,313,908	11.57%
Gypsy/DIRS1	295,497	420,941,275	31.78%
Retroviral	3,033	896,504	0.07%
DNA transposons	62,518	47,030,760	3.55%
Rolling-circles	9,154	9,305,249	0.70%
Unclassified	987,876	353,398,228	26.68%
Total interspersed repeats	62,518	1,028,344,188	77.63%
Small RNA	10,718	3,458,624	0.26%
Satellites	1,677	852,703	0.06%
Simple repeats	186,754	9,448,695	0.71%
Low complexity	26,123	1,422,366	0.11%

Multiple approaches were employed to assess the accuracy and completeness of the genome assembly. First, the genome quality value (QV) was evaluated using the Merqury pipeline (Rhie et al., 2020), confirming high assembly quality. Second, 99.39% of clean PacBio reads aligned to the assembled genome, covering 99.66% of its sequence. Third, CEGMA (Parra et al., 2007) assessment revealed that 90.3% of the 248 Core Eukaryotic Genes (CEGs) were successfully assembled. Finally, BUSCO analysis based on 1, 614 conserved benchmark genes identified 95.7% of Benchmarking Universal Single-Copy Orthologs (95.7% complete, including 94.3% single-copy genes), supporting a high level of genome completeness. Additionally, the GC bias was detected during sequencing (**Table 5**) (**Table 6**).

**Table 5:** BUSCO evaluation of the genome assembly.

BUSCO annotation	Genome assembly	
	Number	Percent
Complete BUSCOs (C)	1522	94.30%
Complete and single-copy BUSCOs (S)	1468	91.00%
Complete and duplicated BUSCOs (D)	54	3.30%
Fragmented BUSCOs (F)	23	1.40%
Missing BUSCOs (M)	69	4.30%
Total BUSCO groups searched	1614	100%

**Table 6:** Estimation of coding genes in the *A. cochinchinensis* genome assembly and genesets using the BUSCO software.

BUSCO annotation	Annotation results	
	Number	Percent
Complete BUSCOs (C)	1498	92.80%
Complete and single-copy BUSCOs (S)	1237	76.60%
Complete and duplicated BUSCOs (D)	261	16.20%
Fragmented BUSCOs (F)	46	2.90%
Missing BUSCOs (M)	70	4.30%
Total BUSCO groups searched	1614	100%

### 3.2 Genome annotation

**Figure 2:**
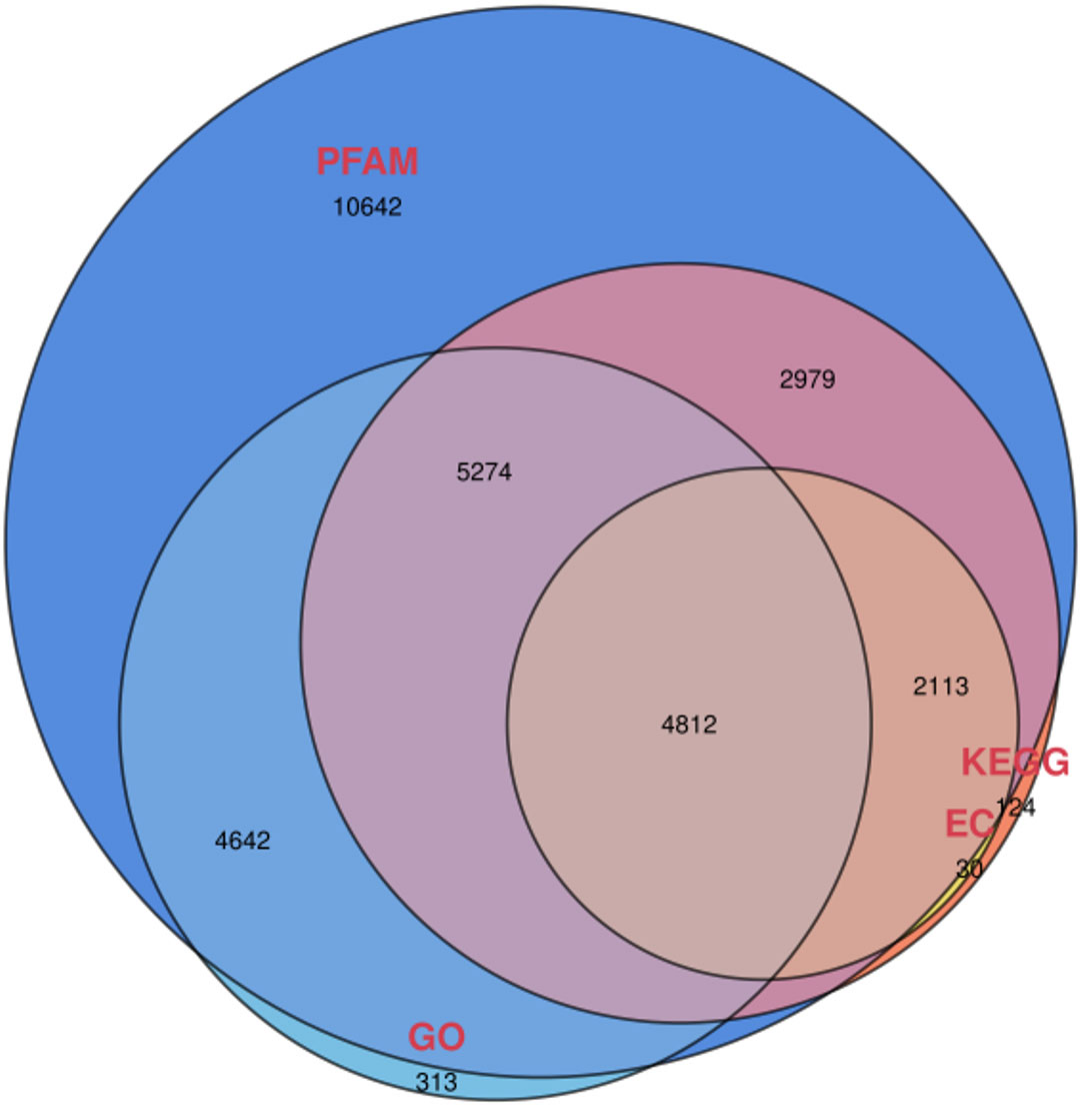
Functional annotation of the *A. cochinchinensis* genome by the PFAM, GO, EC, and KEGG datasets.

Our results indicate that repetitive elements constitute 78.77% of the *A. cochinchinensis* genome. Among these, satellite sequences and transposable elements account for 0.06% and 77.63%, respectively. Within the transposable elements, retroelements represent the predominant type, comprising 47.37% of the genome. Long terminal repeat (LTR) elements alone constitute 43.67%, while DNA transposons represent only 3.55% (**Table 4**). For gene model prediction, we employed a combined approach using *de novo* methods, transcriptomic data, and homology-based strategies. These predictions were integrated into a weighted, non-redundant consensus gene structure using EVidenceModeler (EVM). A total of 48, 453 protein-coding genes were identified, with an average coding sequence (CDS) length of 223.18 bp. These genes were subsequently used for phylogenetic and polysaccharide biosynthesis analyses. Functional annotation was performed by aligning gene sequences against several public databases (eggNOG, PFAM, GO, COG_category, Enzyme Commission number, and KEGG). Of these, 30, 929 genes (63.83%) could be mapped to at least one database, and 4, 812 genes were annotated across all seven databases (**Table 7**; **Figure 2**).

**Table 7:** Statistics of functional annotation of the genome assembly based on six databases.

Database	Number of annotated genes
eggNOG	33,472
PFAM	30,462
GO	15,119
COG_category	31,231
EC	6,980
KEGG	15,410

### 3.3 Comparative genomic analysis

**Figure 3:**
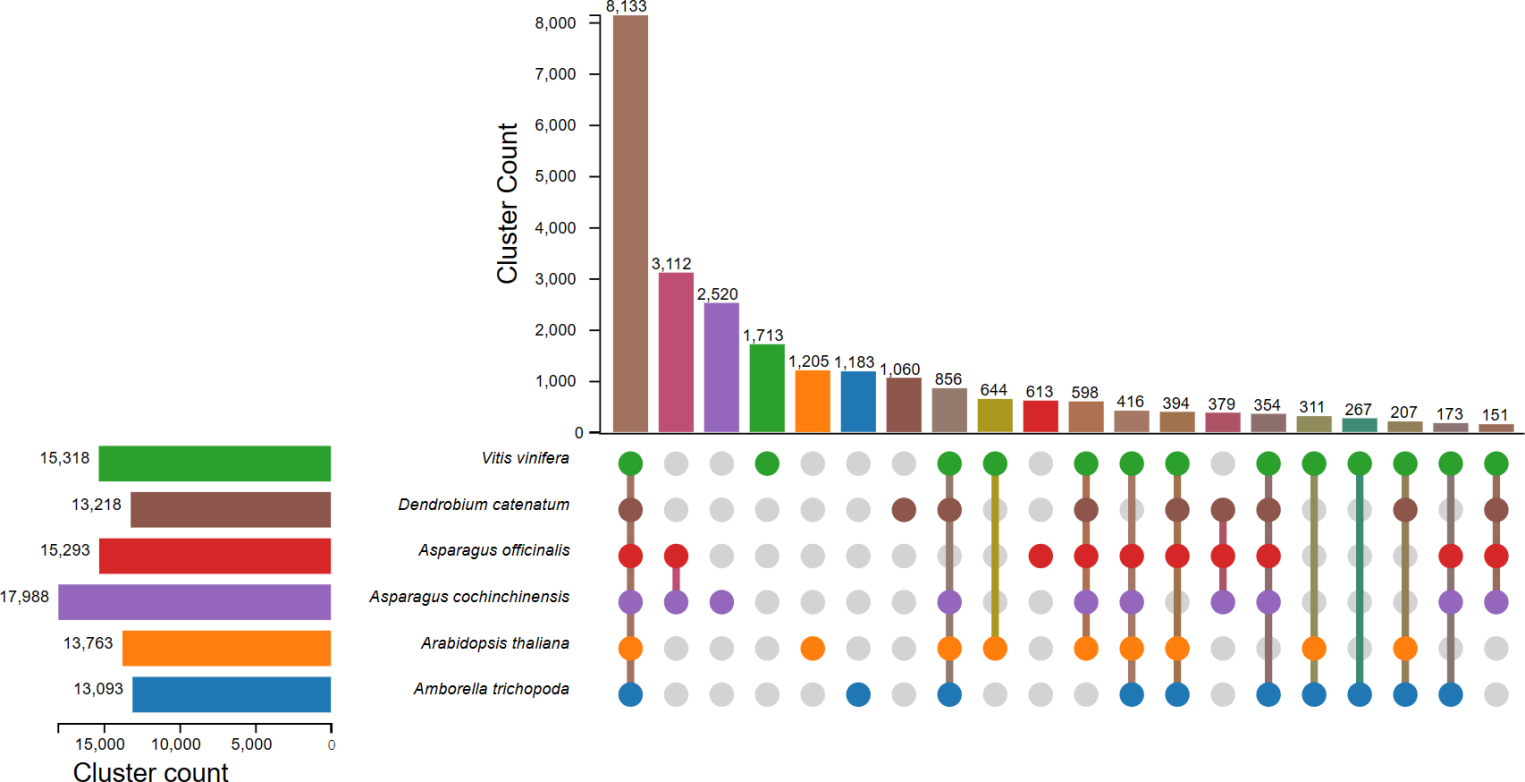
Gene family clustering of *Vitis vinifera*, *Dendrobium catenatum*, *Asparagus officinalis*, *Asparagus cochinchinensis*, *Arabidopsis thaliana*, and *Amborella trichopoda*. Horizontal bar plot (left) displays the distribution of gene cluster counts across each species. Dot plot (bottom-right) illustrates gene cluster sharing among species (horizontal) and comparative groups (vertical), with colored dots indicating shared clusters relative to the species in the bar plot. Vertical bar plot (right) summarizes the distribution of shared and species-specific gene clusters among lineages.

**Figure 4:**
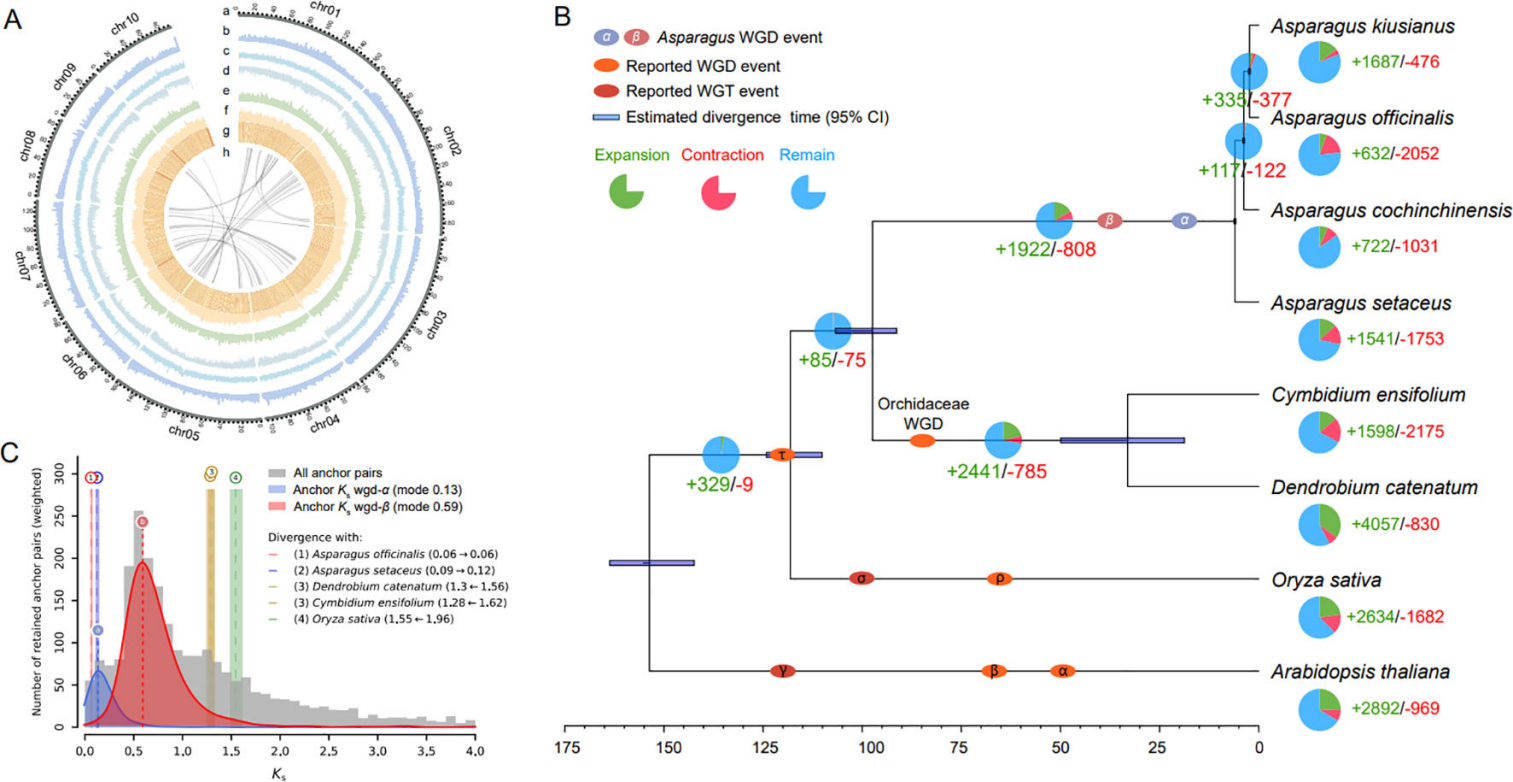
Evolutionary analysis of the *A. cochinchinensis* genome. **(A)**: Characteristics of the 10 chromosomes of *A. cochinchinensis*. a karyotype; b Gene counts; c GC content; d Repeatitive elements content; e Ty1-Copia LTR-RTs content; f Ty3-Gypsy LTR-RTs content; g gene density and h synteny blocks of paralogous sequences of the *A. cochinchinensis* genome. **(B)**: A phylogenetic tree inferred from orthologs of single gene families among selected plant taxa is shown. All nodes received 100% bootstrap support (BS). The pie diagram on each branch of the tree represents the proportion of genes undergoing gain (green) or loss (red) events. The ellipses represent the occurrence of whole genome replication (WGD) events. The estimated divergence time (million years ago, MYA) is indicated at each node; bars are 95% confidence intervals (CI) (each center is defined as mean value). **(C)**: The synonymous substitutions per synonymous site (K_S_) distributions for the anchored paralogs of *A. cochinchinensis* and the anchored orthologs of *A. cochinchinensis*, *A. setaceus*, *D. catenatum*, *C. ensifolium*, and *O. sativa*.

To investigate the evolutionary relationship between *A. cochinchinensis* and other closely related species, we analyzed protein sequences from multiple species, including *Vitis vinifera*, *Amborella trichopoda*, *Asparagus kiusianus*, *Asparagus officinalis*, *Asparagus cochinchinensis*, *Asparagus setaceus*, *Cymbidium ensifolium*, *Dendrobium catenatum*, *Oryza sativa*, and *Arabidopsis thaliana*. Gene family analysis across these species identified a total of 17, 988 gene families, of which 2, 520 were unique to *A. cochinchinensis* (**Figure 3**). A set of 848 single-copy orthologous genes was selected for phylogenetic analysis. The results indicate that the common ancestor of the examined monocots, represented by *O. sativa*, diverged approximately 118 million years ago, while the common ancestor of monocots and dicots—the latter represented here by *A. thaliana*—is estimated to have existed around 154 million years ago. Additionally, asparagus species diverged from the Orchidaceae lineage approximately 95 million years ago. Further divergence time estimates suggest that *A. cochinchinensis* diverged from *A. officinalis* and *A. kiusianus* about 3.77 million years ago, and from *A. setaceus* about 6.02 million years ago (**Figure 4B**).

To compare genomic traits across species, we performed comparative genomic analysis on the eight species using CAFE software. We identified 722 significantly expanded and 1, 031 significantly contracted gene families in the *A. cochinchinensis* genome (*p*< 0.05). KEGG enrichment analysis revealed distinct functional profiles between expanded and contracted gene families. Expanded families were primarily enriched in metabolic pathways and biosynthesis of secondary metabolites, whereas contracted families were mainly associated with ribosome, amino acid biosynthesis, oxidative phosphorylation, and phagosome pathways (**Supplementary Figures 3**, **5**). GO enrichment analysis further showed that expanded gene families were enriched in GO:0055035 (plastid thylakoid membrane), GO:0044391 (ribosomal subunit), and GO:0015934 (large ribosomal subunit), while contracted families were enriched in GO:0016020 (membrane), GO:0071944 (cell periphery), and GO:0005886 (plasma membrane) (**Supplementary Figures 2**, **4**).

To identify genome duplication events and infer evolutionary timelines, we analyzed the synonymous substitution rate (Ks) distribution of homologous gene pairs. In *A. cochinchinensis*, a prominent Ks peak at approximately 0.13 corresponds to a recent whole-genome duplication (WGD) event within the asparagus lineage, while a secondary peak at approximately 0.5 reflects an ancient WGD event shared among monocots. Comparative Ks distributions across species revealed lineage-specific duplication events, including peaks for *A. officinalis* (0.06–0.066), indicating recent divergence within the asparagus genus, and peaks for *D. catenatum* (1.1–1.16) and *C. ensifolium* (1.22–1.26), representing WGD events in Orchidaceae. In *O. sativa*, a Ks peak between 1.5 and 1.96 corresponds to the ancient WGD event in monocots. Two Gaussian distributions fitted to the *A. cochinchinensis* Ks data highlight both recent (~0.13) and ancient (~0.59) duplication events. These findings provide evidence for both recent and ancient duplication events that have shaped the genomic structure of *A. cochinchinensis* and other monocots (**Figure 4C**).

### 3.4 Morphological traits of *Asparagus* root tubers

**Figure 5:**
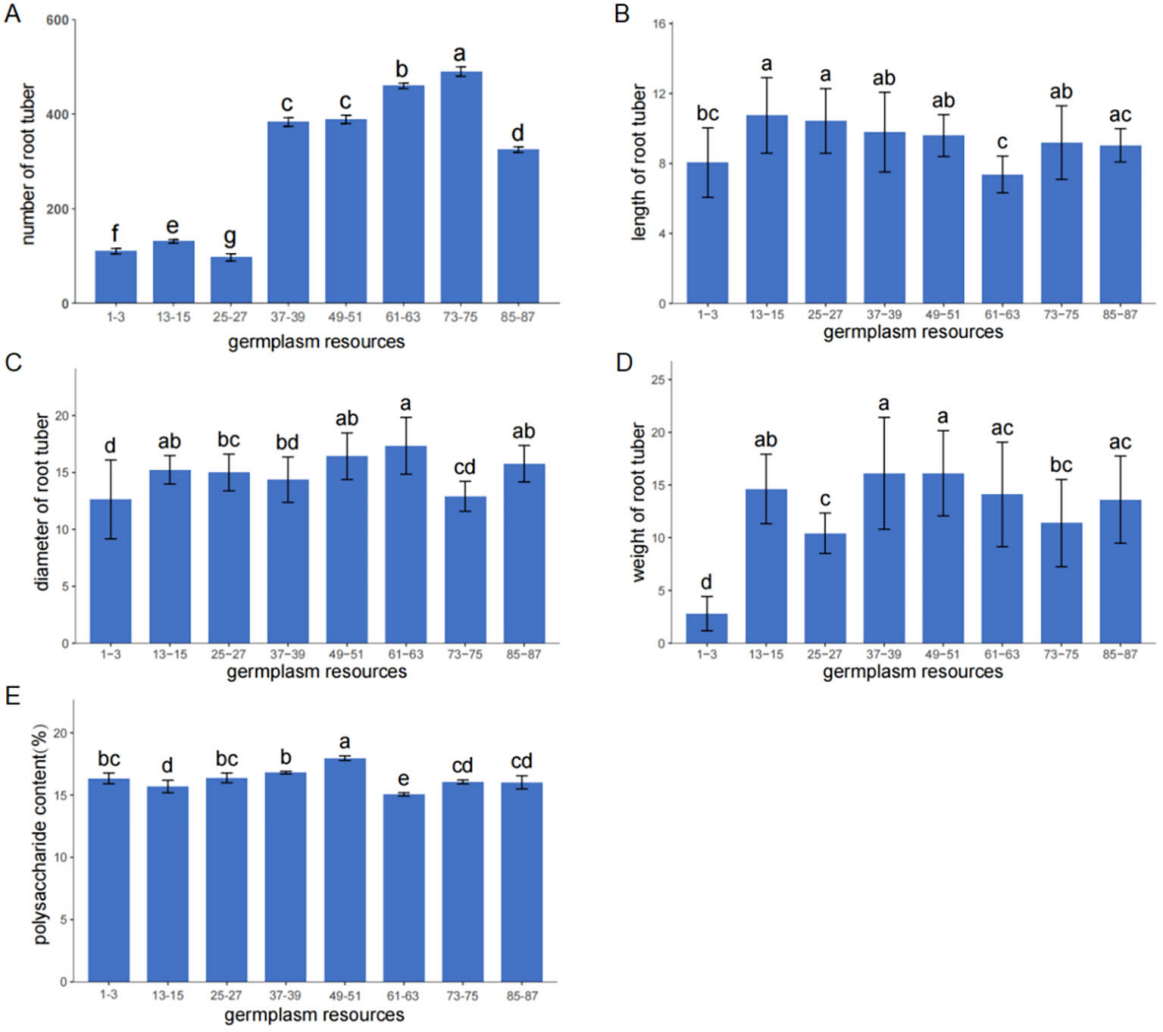
Morphological traits of root tubers from eight genotypes of *A. cochinchinensis*. **(A)** Tuber number per plant. **(B)** Tuber length. **(C)** Tuber diameter. **(D)** Tuber fresh weight. **(E)** Polysaccharide content. Data are presented as mean ± SD (n = 3). Different lowercase letters above the bars indicate statistically significant differences among genotypes as determined by one-way ANOVA followed by LSD test (*p*< 0.05). The corresponding genotype codes and sample numbers are listed in **Table 1**.

In this study, the morphological characteristics of root tubers from eight *Asparagus* species were examined (**Figure 5**). The number of root tubers per plant varied among the species: Accessions Ac4 to Ac8 produced more than 300 tubers each, exceeding the counts observed in Ac1 to Ac3, which is significantly different. Regarding tuber length, no significant differences were detected among Ac2–Ac5, Ac7, and Ac8 (*p*< 0.05), but these groups all exhibited significantly longer tubers than Ac6. Tuber diameter was significantly larger in Ac2, Ac5, Ac6, and Ac8 compared with the other four species. In terms of tuber weight, Ac1 showed the lowest value (2.8 ± 1.6 cm), while Ac2, Ac4, Ac5, Ac6, and Ac8 did not differ significantly from each other but were all markedly heavier than Ac1 (2.8 ± 1.6 cm). Polysaccharide content in root tubers also differed clearly among species: Ac5 contained the highest polysaccharide level (17.96 ± 0.18%), exceeding that of the other seven accessions, whereas Ac6 displayed the lowest content. The present study focuses on polysaccharide content in the root tubers.

### 3.5 Genes that involved in *A. cochinchinensis* polysaccharides biosynthesis

**Figure 6:**
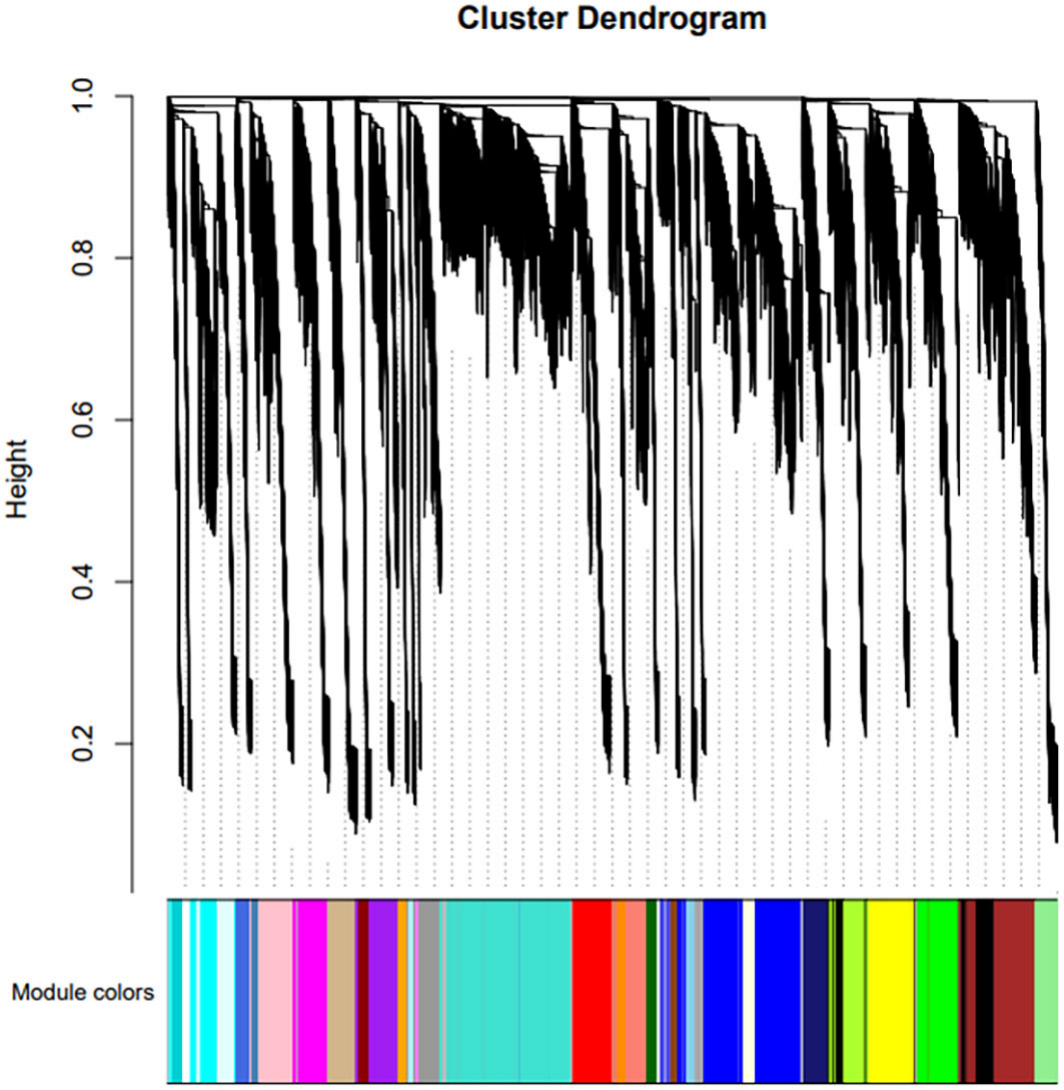
Gene co-expression clustering and module identification. This clustering dendrogram was generated by WGCNA. It shows the hierarchical clustering of all detected genes based on their topological overlap in expression patterns across samples. Genes with similar expression profiles are clustered together and assigned to different co-expression modules, each represented by a distinct color block below the dendrogram. Genes within the same module are likely functionally related. Modules whose expression profiles correlate strongly with polysaccharide content (identified in **Figure 7**) are considered candidates for containing key genes regulating polysaccharide biosynthesis in *A. cochinchinensis*.

**Figure 7:**
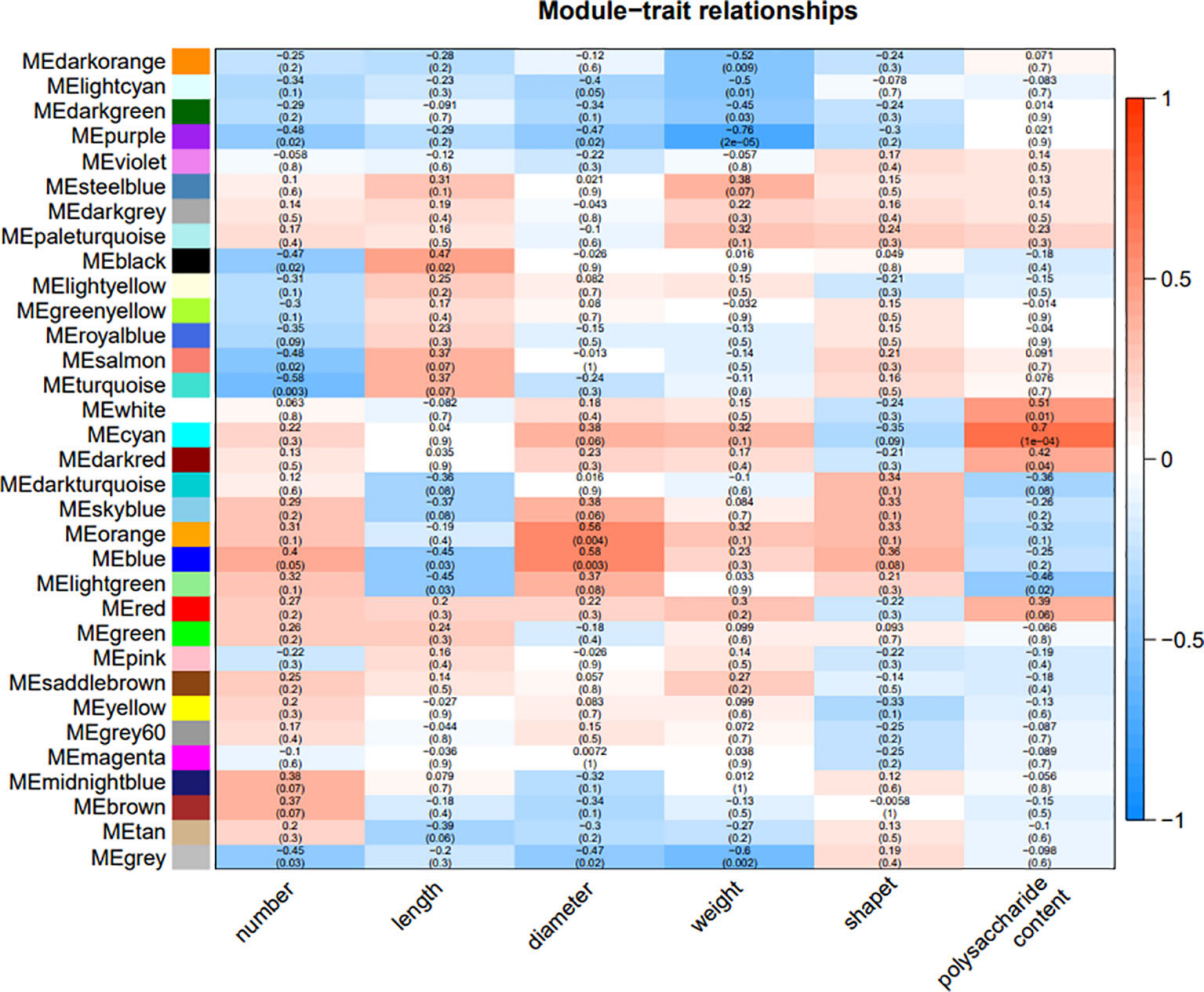
Module-trait associations. Association between gene modules and phenotypic traits.​ Color-coded gene modules identified by WGCNA are labeled on the left. Heatmap (right) depicts Pearson correlation coefficients between modules and traits, color-graded from red (maximum positive correlation, +1) to blue (maximum negative correlation, –1). Numerical values and corresponding p-values (in parentheses) are indicated within each cell.

Weighted Gene Co-expression Network Analysis (WGCNA) revealed associations between co-expression modules and key phenotypic traits of *A. cochinchinensis*, including root tuber number, length, diameter, weight, shape, and polysaccharide content (**Figures 6**, **7**). Root tuber number per plant exhibited the strongest negative correlation with the MEturquoise module (r = −0.58, *p* < 0.01). Tuber diameter showed significant negative correlations with the MEblue (r = −0.58, *p* < 0.01) and MEorange (r = −0.56, *p* < 0.01) modules. Tuber weight was highly negatively correlated with the MEpurple (r = −0.76, p < 0.001) and MEdarkorange (r = −0.52, *p* < 0.01) modules. Polysaccharide content was positively correlated with the MEwhite (r = 0.51, *p* < 0.05) and MEcyan (r = 0.70, *p* < 0.001) modules, which containing genes participating in carbon and sugar metabolism, suggesting their potential role in regulating this trait. Additionally, the MElightgreen module displayed a weaker but still significant negative correlation with polysaccharide content (r = −0.46, *p* < 0.05). In contrast, no module showed a strong correlation (|r| > 0.5) with either tuber length or shape. Overall, these results demonstrate statistically significant associations between specific co-expression modules and key phenotypic traits, revealing a modular genetic architecture underlying tuber development and polysaccharide accumulation in *A. cochinchinensis*.

**Figure 8:**
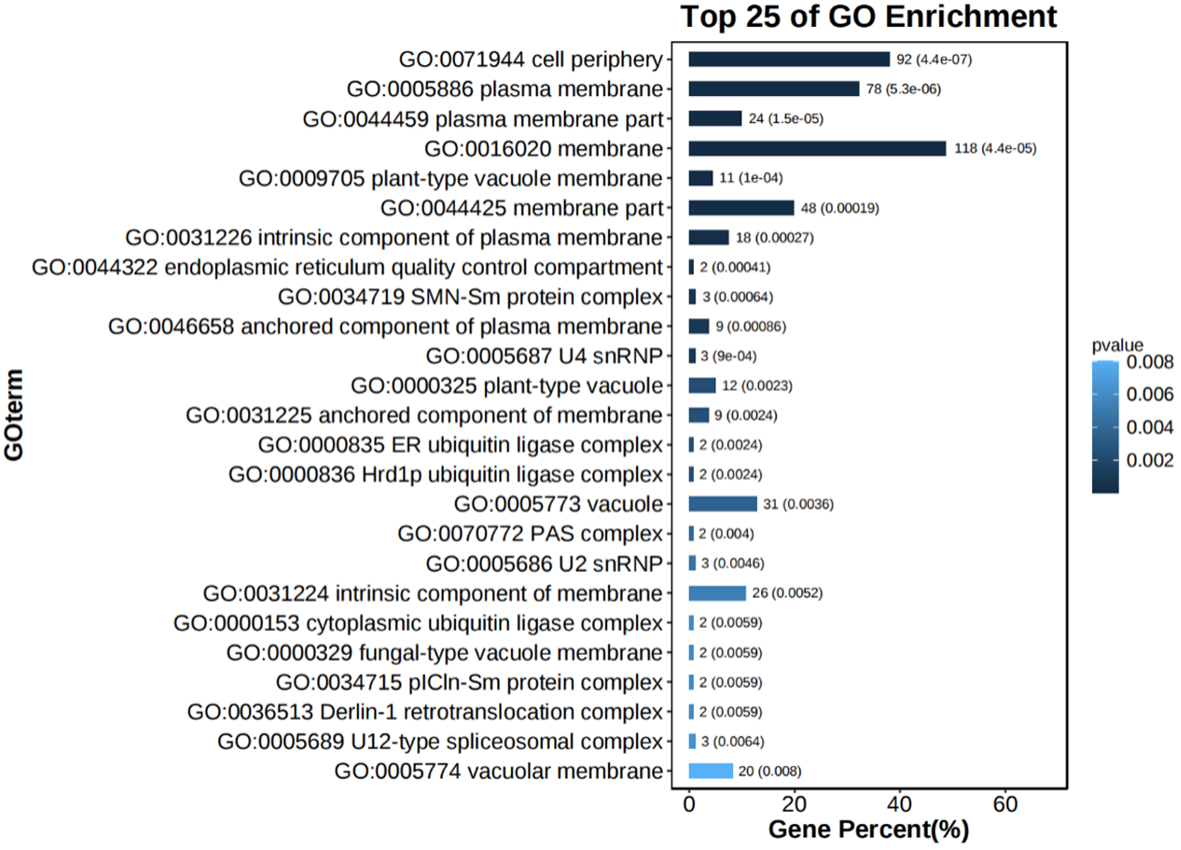
Top 25 GO enrichment terms of the DEGs between WGCNA and root tuber upregulated genes.

**Figure 9:**
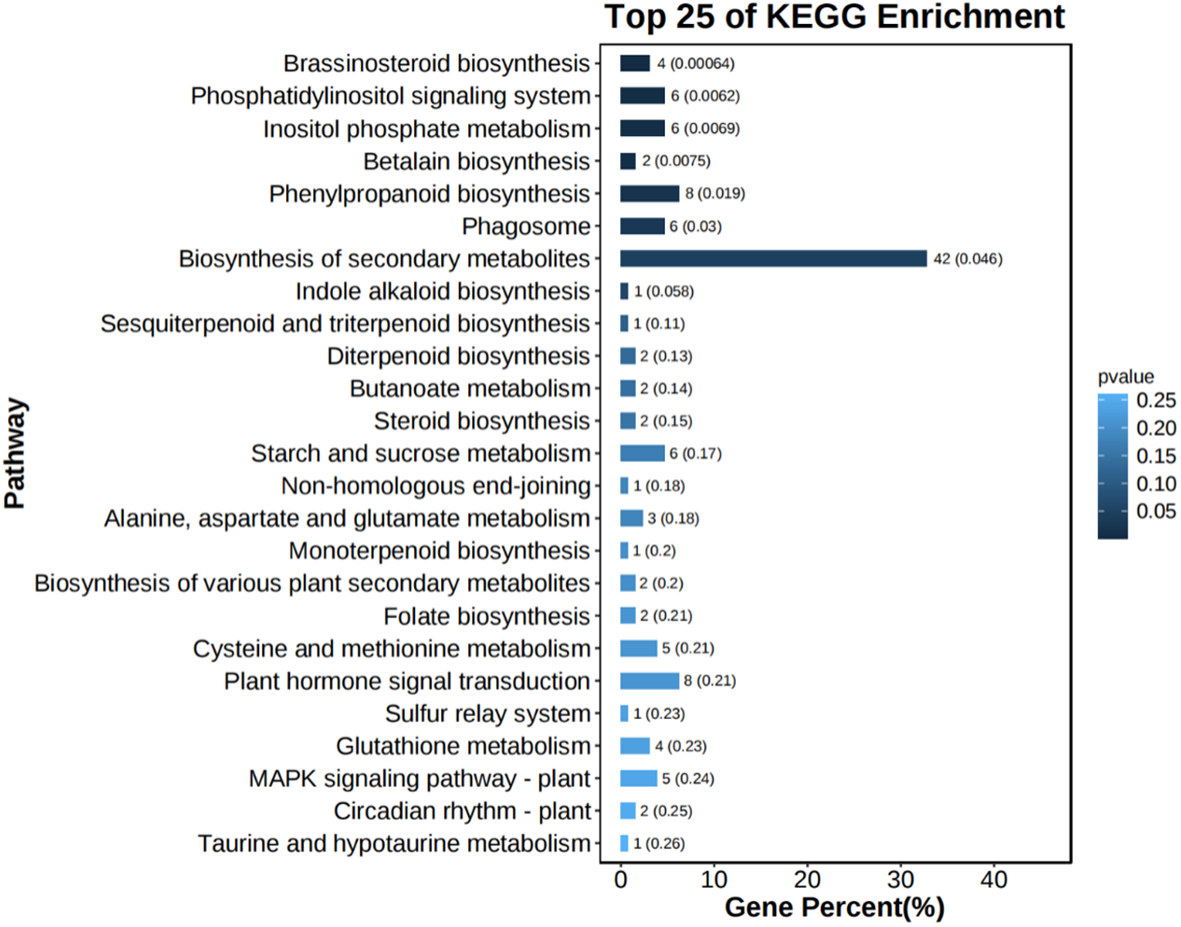
Top 25 pathways in the KEGG enrichment analysis of DEGs between WGCNA and root tuber upregulated genes.

WGCNA identified four modules positively correlated with polysaccharide content, encompassing 4, 158 genes. Additionally, 8, 521 differentially expressed genes (DEGs) were found to be up-regulated in root tubers compared to other tissues. A Venn analysis of these two gene sets yielded 729 overlapping genes (**Supplementary Figure 6**). GO and KEGG enrichment analyses of these common genes revealed that they were primarily enriched in membrane-related GO terms, including GO:0016020 (membrane), GO:0071944 (cell periphery), GO:0005886 (plasma membrane), and GO:0044425 (membrane part) (**Figure 8**). KEGG analysis showed that the majority (42%) of common genes were enriched in Biosynthesis of secondary metabolites (ko01110), followed by Phenylpropanoid biosynthesis (ko00940), Phosphatidylinositol signaling system (ko04070), and Inositol phosphate metabolism (ko00562) (**Figure 9**).

From the 8, 521 root-tuber up-regulated DEGs, we further identified genes specifically up-regulated in the root tuber of accession Ac5, which exhibits the highest polysaccharide content. This analysis yielded 118 Ac5-specific up-regulated genes. GO and KEGG enrichment analyses of these genes indicated that the most enriched KEGG pathways were Metabolic pathways and Biosynthesis of secondary metabolites (each containing 10 genes) (**Supplementary Figure 8**). Notably, two genes, Aochr07G000468 and Aochr07G000469, were enriched in the Starch and sucrose metabolism pathway, suggesting a potential role in polysaccharide biosynthesis and accumulation in *A. cochinchinensis* root tubers. GO enrichment analysis of Ac5-specific genes showed enrichment in terms such as GO:0009506 (plasmodesma), GO:0055044 (symplast), GO:0005911 (cell–cell junction), GO:0030054 (cell junction), and GO:0005730 (nucleolus) (**Supplementary Figure 7**).

### 3.6 Construction of polysaccharide biosynthesis pathways in root tuber and fibrous root of *A. cochinchinensis*

To construct the polysaccharide biosynthesis pathways in root tuber and fibrous root of *A. cochinchinensis*, heat maps were generated based on FPKM values from transcriptomic data to visualize the expression levels of genes involved in polysaccharide biosynthesis. The pathway consists of three sequential stages: sucrose conversion, monosaccharide activation, and polysaccharide polymerization.

**Figure 10:**
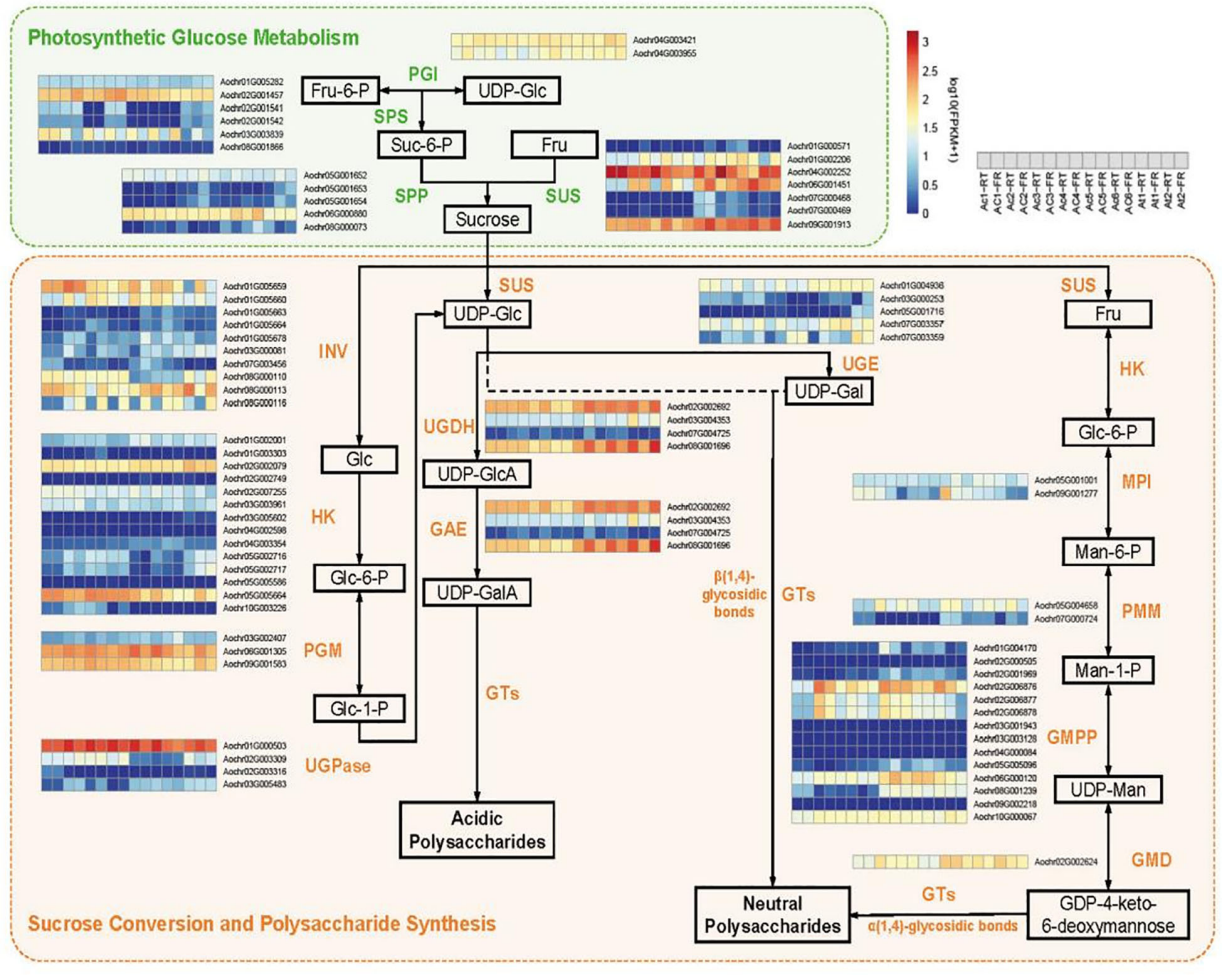
Construction of the polysaccharide biosynthesis pathway using Microsoft PowerPoint 2019. The DEGs encoding enzymes in the pathway are shown. The columns and rows indicate samples and genes, respectively. Fru-6-P, Fructose-6-Phosphate; PGI, Phosphoglucose Isomerase; UDP-Glc, Uridine Diphosphate Glucose; SPS, Sucrose Phosphate Synthase; Suc-6-P, Sucrose-6-Phosphate; Fru, Fructose; SPP, Sucrose Phosphatase; SUS, Sucrose Synthase; INV, Invertase; Glc, Glucose; HK, Hexokinase; Glc-6-P, Glucose-6-Phosphate; PGM, Phosphoglucomutase; Glc-1-P, Glucose-1-Phosphate; UGPase, Uridine Diphosphate-Glucose Pyrophosphorylase; UGDH, Uridine Diphosphate-Glucose Dehydrogenase; UDP-GlcA, Uridine Diphosphate Glucuronic Acid; GAE, Uridine Diphosphate-D-Glucuronate 4-Epimerase; UDP-GalA, Uridine Diphosphate Galacturonic Acid; UGE, Uridine Diphosphate-Glucose 4-Epimerase; UDP-Gal, Uridine Diphosphate Galactose; MPI, Mannose-6-Phosphate Isomerase; Man-6-P, Mannose-6-Phosphate; PMM, Phosphomannomutase; Man-1-P, Mannose-1-Phosphate; GMPP, Guanosine Diphosphate-Mannose Pyrophosphorylase; UDP-Man, Uridine Diphosphate Mannose; GMD, Guanosine Diphosphate-Mannose 4,6-Dehydratase; GDP-4-keto-6-deoxymannose, Guanosine Diphosphate-4-Keto-6-Deoxymannose; GTs, Glycosyltransferases; FR, Fibrous Root; RT, Root Tuber.

Based on the transcript profiles in root tuber and fibrous root, sixteen genes were identified as highly expressed in both tissues and were classified into different gene families corresponding to each biosynthetic stage (**Figure 10**). At the sucrose conversion stage, one SucrosePhosphate Synthase (SPS) gene (Aochr02G001457), one Sucrose Phosphatase (SPP) gene (Aochr06G000880), and three Sucrose Synthase (SUS) genes (Aochr04G002252, Aochr06G001451, Aochr09G001913) showed high expression; at the monosaccharide activation stage, seven highly expressed genes were identified, including two Invertase (INV) genes (Aochr01G005659, Aochr08G000113), two Hexokinase (HK) genes (Aochr02G002079, Aochr05G005664), two Phosphoglucomutase (PGM) genes (Aochr06G001305, Aochr09G001583), and one Uridine Diphosphate-Glucose Pyrophosphorylase (UGPase) gene (Aochr01G000503); at the polysaccharide polymerization stage, four highly expressed genes were identified, comprising two Uridine Diphosphate-Glucose Dehydrogenase (UGDH) genes (Aochr02G002692, Aochr08G001696) and two GuanosineDiphosphate-Mannose Pyrophosphorylase (GMPP) genes (Aochr02G006876, Aochr06G000120). All of the above highly expressed genes in root tuber and fibrous root are likely involved in polysaccharide biosynthesis in *A. cochinchinensis*.

## 4 Discussion

### 4.1 Genome assembly, repetitive sequences and evolutionary dynamics

*Asparagus cochinchinensis* is a widely utilized medicinal plant with ornamental value. It is closely related to *A. officinalis*, an important vegetable crop, and *A. setaceus*, a popular ornamental species. Comparative genetic and genomic studies of these *Asparagus* species are therefore crucial for understanding the molecular mechanisms underlying important agricultural traits, the biosynthesis of active compounds, and their evolutionary origins. However, research on *A. cochinchinensis* remains limited, with few molecular-level studies conducted to date. A high-quality genome sequence would greatly advance studies on this species and facilitate comparative analysis within the genus.

Many plant genomes exhibit high heterozygosity and repetitive content, which complicate genome assembly. To overcome these challenges, we employed an integrated sequencing strategy for *A. cochinchinensis*, combining Illumina short reads, PacBio HiFi long reads, and Hi-C sequencing data. The primary assembly was generated using PacBio HiFi reads, followed by polishing with accurate short reads. Hi-C data were then used for scaffolding and chromosome-level assembly. This approach produced a chromosome-scale genome assembly with high completeness and accuracy. Benchmarking analysis with BUSCO revealed that our assembly exhibited a completeness of 94.3% for the set of universal single-copy orthologs. This proportion is lower than the 96.1% reported for *Osmanthus fragrans* (Yang et al., 2018) and 96.77% for *Brassica oleracea* (Sun et al., 2019), but higher than the scores for *Ginkgo biloba* (73.95%) (Guan et al., 2016), *Asparagus officinalis* (88.2%) (Harkess et al., 2017), and *Asparagus setaceus* (90.0%) (Harkess et al., 2017). Given the high heterozygosity and repetitive nature of the genome, the assembly presented here represents a high-quality genomic resource for *A. cochinchinensis*.

To ensure accurate genome annotation, we applied multiple methods for protein-coding gene prediction and used an integrated pipeline to analyze repetitive sequences. The majority of genes were functionally annotated. Repetitive sequences, predominantly retroelements, constitute a major component of eukaryotic genomes and play important roles in genome evolution (Wicker et al., 2018; Samoluk et al., 2019), chromosome rearrangement (Li et al., 2017), and gene regulation (Uzunović et al., 2019). Repetitive sequences accounted for 78.77% of the *A. cochinchinensis* genome, suggesting their potential significance in the evolution and domestication of this species.

Our findings provide valuable insights into the genome structure, gene family evolution, and expression profiles of candidate genes associated with polysaccharide accumulation in *A. cochinchinensis*, illuminating its unique biological characteristics and adaptive strategies. The successful assembly of a high-quality reference genome represents a significant achievement. The application of multiple evaluation metrics, such as GC content and BUSCO completeness, confirms the robustness and reliability of the assembly. This genomic resource will support investigations into the evolution of the *Asparagus* genus and genetic variation in polysaccharide biosynthetic pathways, thereby guiding future domestication efforts aimed at enhancing medicinal efficacy.

Whole-genome duplication (WGD) events have played a major role in genome and gene evolution across plants. Previous studies have shown lineage-specific WGD patterns; for example, grape did not undergo additional WGD after the ancient γ-event shared by eudicots, while tea experienced two additional rounds (Wei et al., 2018). Monocots share an ancestral WGD, after which lineage-specific events occurred (Ming et al., 2015). Earlier work indicated that *A. officinalis* underwent at least two ancient WGDs prior to its divergence from other asparagus species (Harkess et al., 2017). In this study, Ks distribution analysis revealed two distinct peaks in *A. cochinchinensis*, corresponding to two WGD events that occurred later than those in *A. officinalis* and *A. setaceus*. These WGD events and subsequent diploidization have significantly shaped the present genome structure of *A. cochinchinensis*.

### 4.2 Expression profiles of candidate carbohydrate metabolism genes associated with polysaccharide accumulation

A total of 48, 453 protein-coding genes were predicted in the *A. cochinchinensis* genome, providing a valuable resource for comparative genomics and evolutionary studies within the genus, as well as critical information for breeding programs. This high-quality reference genome will enable the identification of genes associated with important agronomic traits, including the biosynthesis and accumulation of active compounds and end-use functionality. These genomic resources are essential for the genetic improvement of *A. cochinchinensis*.

Polysaccharides are major bioactive components in *A. cochinchinensis* root tubers, determining their quality and medicinal efficacy. However, the genetic basis and key candidate genes associated with polysaccharide accumulation in *A. cochinchinensis* remain poorly understood, limiting further research and comprehensive utilization of this species. To address this gap, we combined genome sequencing and transcriptomic analysis. Plant polysaccharide biosynthesis is a complex process involving numerous genes and enzymes (Zabotina et al., 2021). Previous studies in other species have identified key genes involved in polysaccharide biosynthesis, such as AXS, GMPP, and GALE in *Polygonatum* species (Lu et al., 2023); DoHY5, DoF3H1, DoGMPP2, and DoPMT28 in *Dendrobium officinale* (Li et al., 2023), as well as genes catalyzing sucrose, glucose-6P, and mannose-6P synthesis in *Bletilla striata* (Xu et al., 2024). In this study, we identified and analyzed genes involved in polysaccharide biosynthesis in *A. cochinchinensis*, especially two candidate genes, Aochr07G000468 and Aochr07G000469, which may be closely associated with polysaccharide accumulation in root tubers. These findings enhance our understanding of polysaccharide metabolism in *A. cochinchinensis* and provide a foundation for functional studies and molecular breeding.

Polysaccharides in *A. cochinchinensis* serve as non-specific immuno-enhancers that boost host immune responses (Zhang et al., 2018). They consist mainly of acidic heteropolysaccharides with minor neutral polysaccharides, containing monosaccharides such as galactose, glucose, mannose, glucuronic acid, and galacturonic acid (Koo et al., 2000; Xu et al., 2025). To elucidate their biosynthesis, we identified polysaccharide metabolism-related genes from transcriptomic data and reconstructed the biosynthetic pathways in root tuber and fibrous root tissues. The pathway comprises three sequential stages: sucrose conversion, monosaccharide activation, and polysaccharide polymerization.

In the first stage, photosynthetic products serve as initial substrates. Sucrose phosphate synthase (SPS) catalyzes the formation of sucrose-6-phosphate from UDP-glucose and fructose-6-phosphate, which is dephosphorylated by sucrose-phosphate phosphatase (SPP) to yield sucrose. Alternatively, sucrose synthase (SUS) directly synthesizes sucrose from UDP-glucose and fructose (Stein and Granot, 2019). High expressions of one SPS gene (Aochr02G001457), one SPP gene (Aochr06G000880), and three SUS genes (Aochr04G002252, Aochr06G001451, Aochr09G001913) indicate their roles in directing photosynthetic products toward sucrose production.

In the second stage, sucrose is cleaved by invertase (INV) and SUS into glucose, UDP-glucose, and fructose (Li et al., 2023). Glucose is converted to UDP-glucose via hexokinase (HK), phosphoglucomutase (PGM), and UDP-glucose pyrophosphorylase (UGPase) (Yang et al., 2013; Wang et al., 2017). Highly expressed genes in this stage include two INV genes (Aochr01G005659, Aochr08G000113), two HK genes (Aochr02G002079, Aochr05G005664), two PGM genes (*Aochr06G001305*, *Aochr09G001583*), and one UGPase gene (Aochr01G000503).

In the third stage, UDP-glucose is converted to UDP-glucuronic acid by UDP-glucose dehydrogenase (UGDH) and to UDP-galactose by UDP-glucuronic acid epimerase (UGE) (Klinghammer and Tenhaken, 2007; Reboul et al., 2011). UDP-glucuronic acid is further epimerized to UDP-galacturonic acid (Gu and Bar-Peled, 2004). Fructose-derived mannose-6-phosphate is converted to GDP-mannose via phosphomannomutase (PMM) and GDP-mannose pyrophosphorylase (GMPP) (Qian et al., 2007). High expressions of two UGDH genes (Aochr02G002692, Aochr08G001696) and two GMPP genes (Aochr02G006876, Aochr06G000120) highlight their importance in activated sugar donor production. Finally, glycosyltransferases polymerize these activated sugars into acidic and neutral polysaccharides. The polysaccharide biosynthetic pathway constructed in this study, along with the key genes identified, establishes a solid foundation for future functional validation and molecular breeding of *A. cochinchinensis*.

## 5 Conclusion

In the present study, we assembled a high-quality chromosome-level genome for *A. cochinchinensis* by combining data from three sequencing technologies: Illumina short reads, PacBio HiFi, and Hi-C. The resulting assembly spans 1.52 Gb, consisting of 145 scaffolds with an N50 of 157.6 Mb. We annotated 48, 453 protein-coding genes, with an average coding sequence (CDS) length of 223.18 bp, among which 4, 812 genes were annotated in all seven databases used. Comparative genomic analysis revealed the divergence time between *A. cochinchinensis* and closely related species. Two unique WGD events were identified during its evolutionary history. Furthermore, we constructed an expression atlas of candidate carbohydrate metabolism genes in root tuber and fibrous root tissues of *A. cochinchinensis* and identified key candidate genes associated with polysaccharide accumulation. In total, sixteen genes across different gene families were found to be closely associated with polysaccharide biosynthesis in root tubers. The newly assembled genome and the identified polysaccharide biosynthesis genes provide a valuable genomic resource for future biological, ecological, and breeding studies of *A. cochinchinensis*. This resource will support molecular breeding efforts aimed at improving traits such as yield, growth rate, and polysaccharide content.

## Data Availability

The genomic data that support the findings of this study have been deposited into CNSA with accession number CNP0009705.

## Supplementary material

Supplementary Figure 1 Evaluation of *A. cochinchinensis* genome characteristics based on k-mer analysis.​ The plot shows k-mer frequency (Y-axis) versus coverage depth (X-axis). The blue curve represents the observed k-mer distribution, while the black curve denotes the full model fitted by GenomeScope. The yellow and orange curves correspond to the unique sequence and error distributions, respectively. Vertical dashed lines mark integer multiples of the predicted primary peak coverage.

Supplementary Figure 2 Top 25 GO enrichment terms of the expanded gene families in *A. cochinchinensis* genome.

Supplementary Figure 3 Top 25 pathways in the KEGG enrichment analysis of expanded gene families.

Supplementary Figure 4 Top 25 GO enrichment terms of the contracted gene families in *A. cochinchinensis* genome.

Supplementary Figure 5 Top 25 pathways in the KEGG enrichment analysis of contrancted gene families.

Supplementary Figure 6 Venn diagram of common DEGs between WGCNA and root tuber upregulated genes. Venn diagram shows the intersection of critical signatures obtained by the two strategies.

Supplementary Figure 7 Top 25 GO enrichment terms of the specially upregulated DEGs in the Ac5 root tuber.

Supplementary Figure 8 Top 25 pathways in the KEGG enrichment analysis of the specially upregulated DEGs in the Ac5 root tuber.
