# Supplementary material for: Chromosome-scale genome assembly, carbohydrate metabolism transcriptome atlas, and candidate genes for polysaccharide accumulation in Asparagus cochinchinensis
Source: Front Plant Sci. 2026 Jun 29;17:1867413. doi: 10.3389/fpls.2026.1867413 (PMC13357925; doi:10.3389/fpls.2026.1867413)
Supplement: Supplementary Figure 1 — Evaluation of A. cochinchinensis genome characteristics based on k-mer analysis.​ The plot shows k-mer frequency (Y-axis) versus coverage depth (X-axis). The blue curve represents the observed k-mer distribution, while the black curve denotes the full model fitted by GenomeScope. The yellow and orange curves correspond to the unique sequence and error distributions, respectively. Vertical dashed lines mark integer multiples of the predicted primary peak coverage. [file Table1.docx]

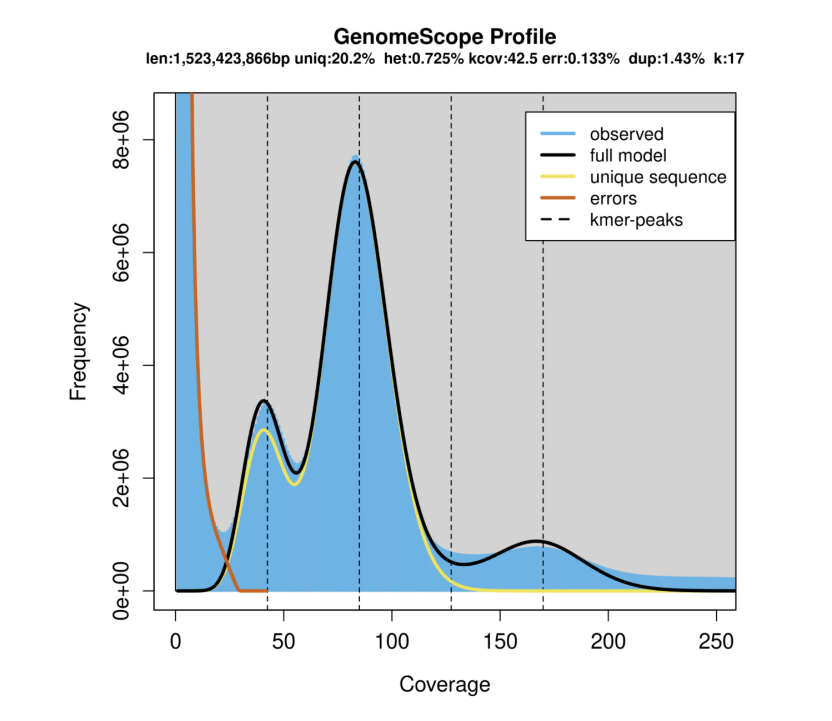


**Figure S1. Evaluation of *A. cochinchinensis* genome characteristics based on k-mer analysis.​** The plot shows k-mer frequency (Y-axis) versus coverage depth (X-axis). The blue curve represents the observed k-mer distribution, while the black curve denotes the full model fitted by GenomeScope. The yellow and orange curves correspond to the unique sequence and error distributions, respectively. Vertical dashed lines mark integer multiples of the predicted primary peak coverage.


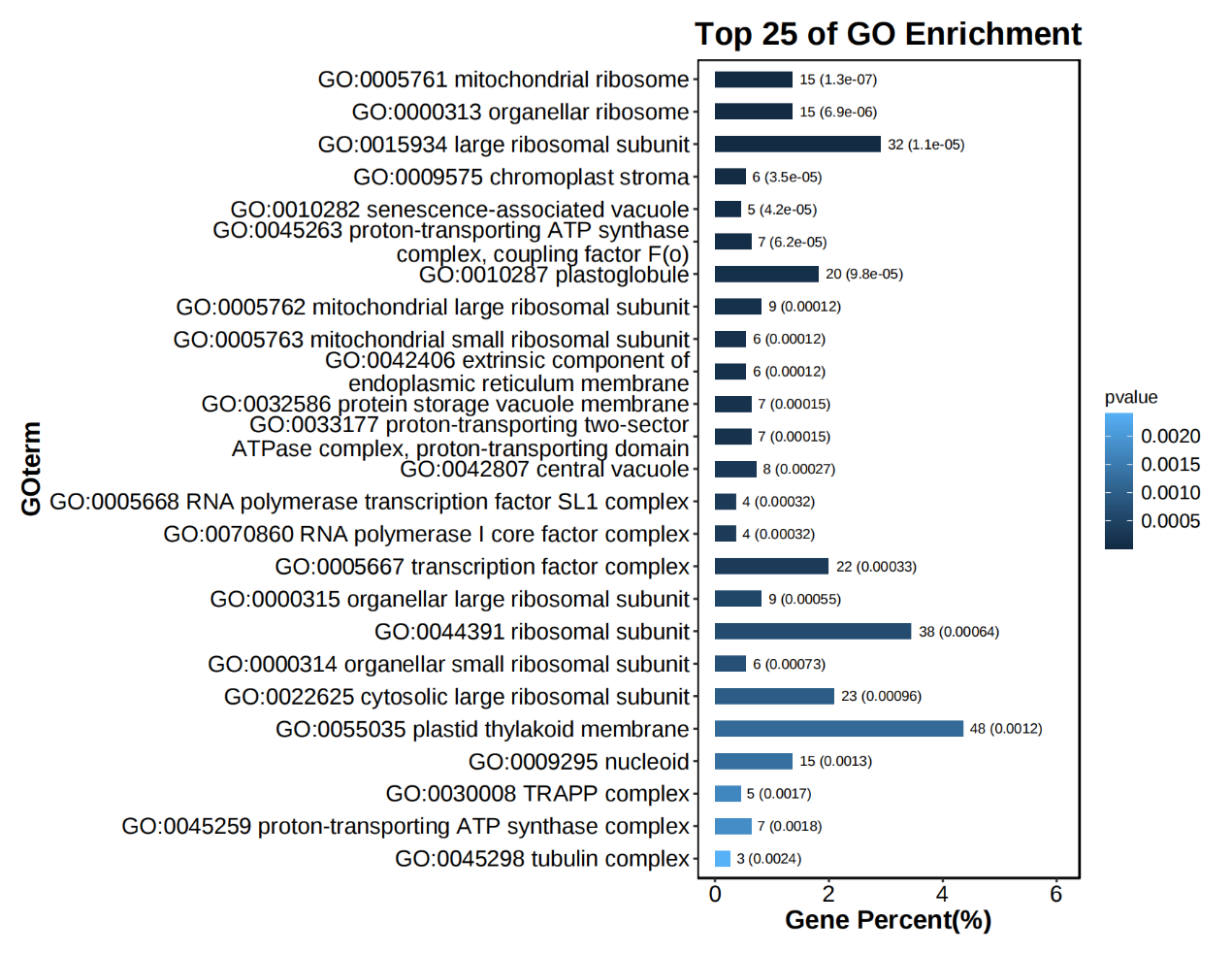


**Figure S2. Top 25 GO enrichment terms of the expanded gene families in *A. cochinchinensis* genome.**


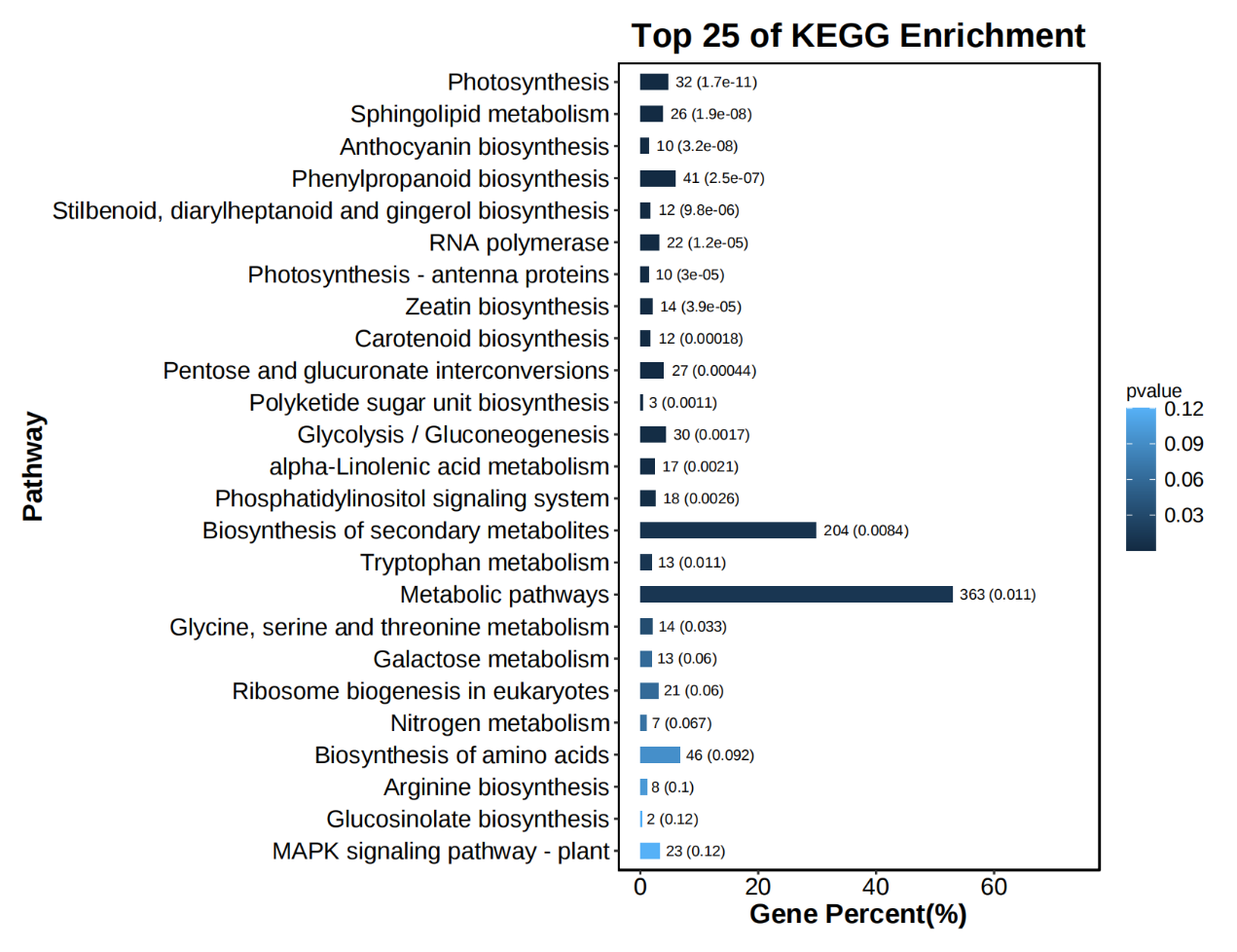


**Figure S3. Top 25 pathways in the KEGG enrichment analysis of expanded gene families.**


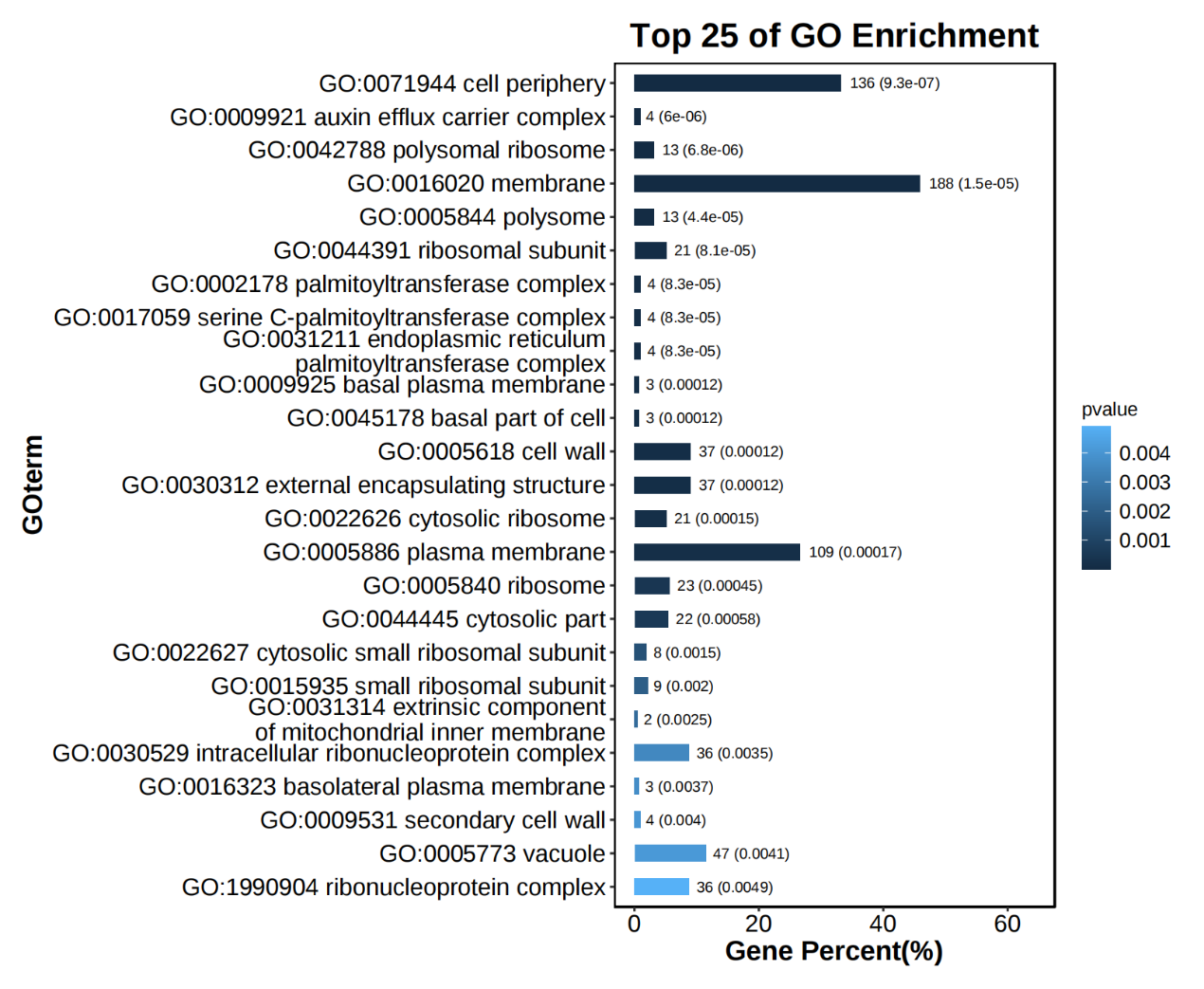


**Figure S4. Top 25 GO enrichment terms of the contracted gene families in *A. cochinchinensis* genome.**


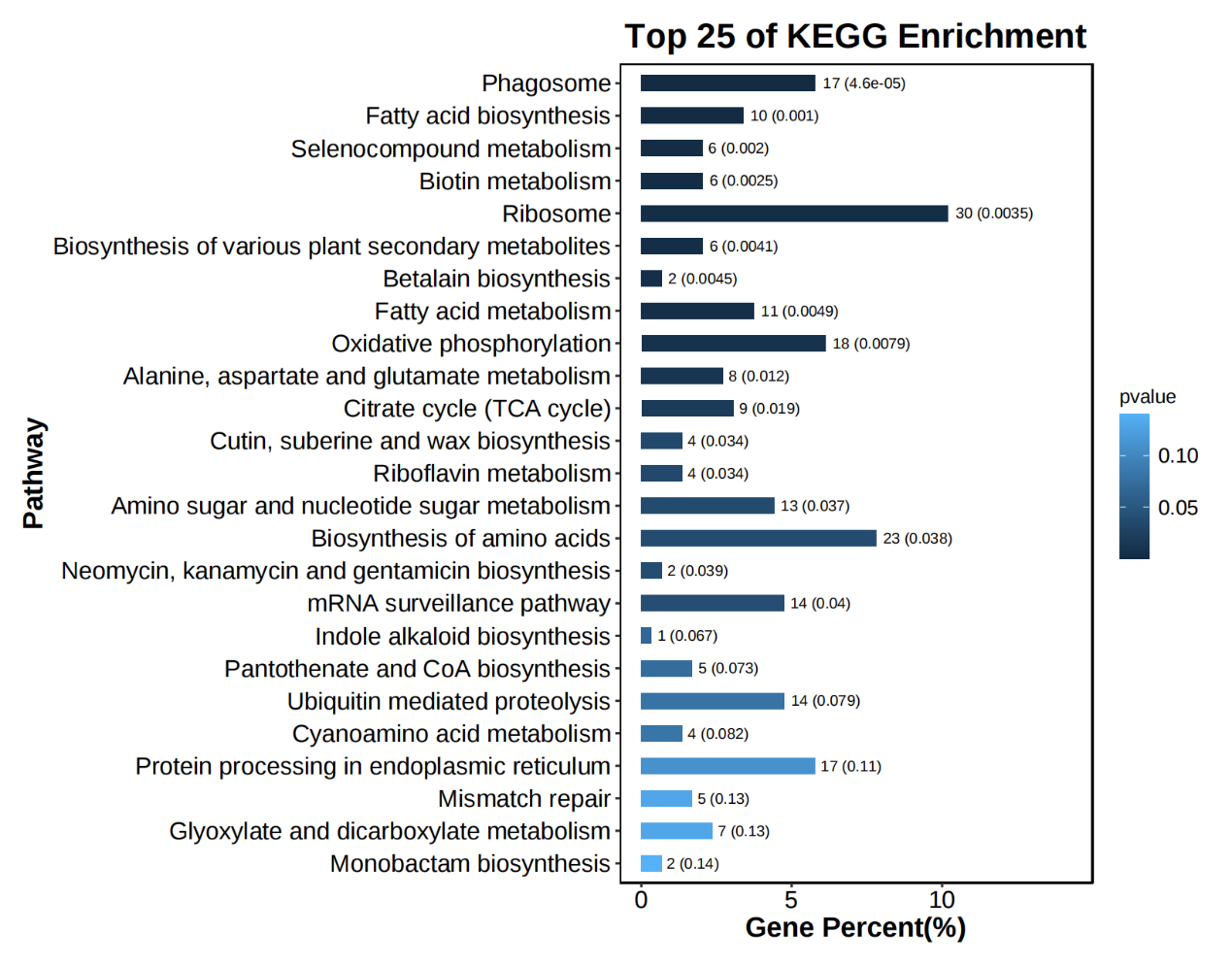


**Figure S5. Top 25 pathways in the KEGG enrichment analysis of contrancted gene families.**


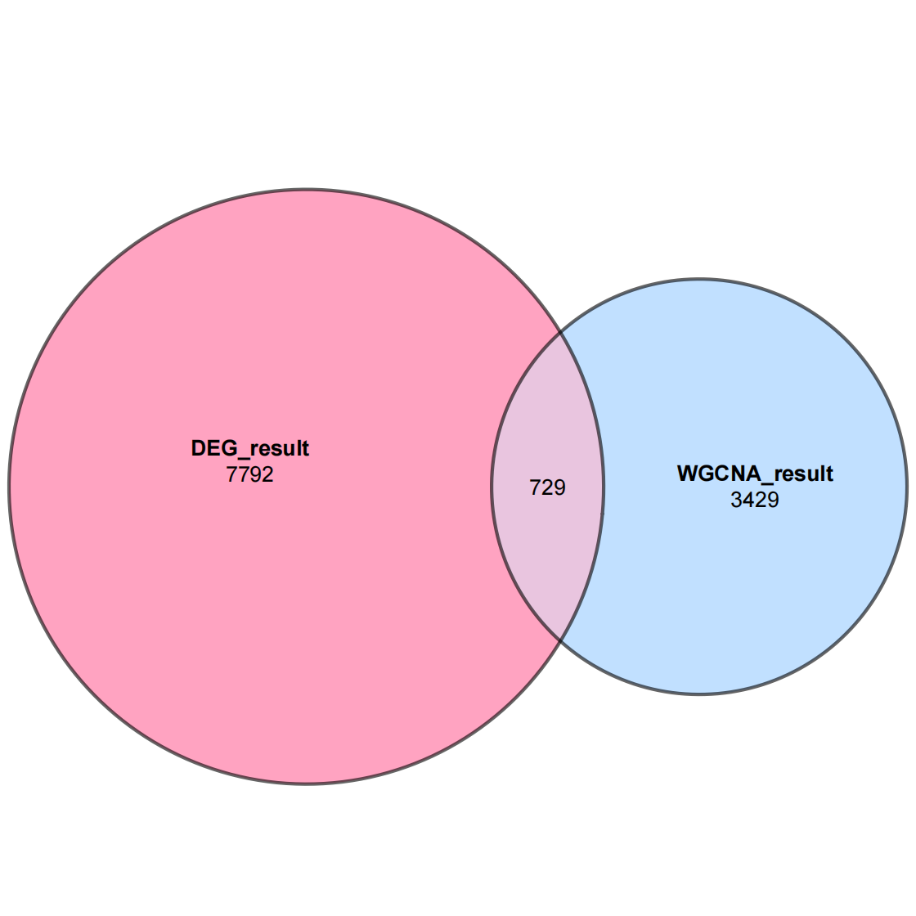


**Figure S6. Venn diagram of common DEGs between WGCNA and root tuber upregulated genes.** Venn diagram shows the intersection of critical signatures obtained by the two strategies.

**
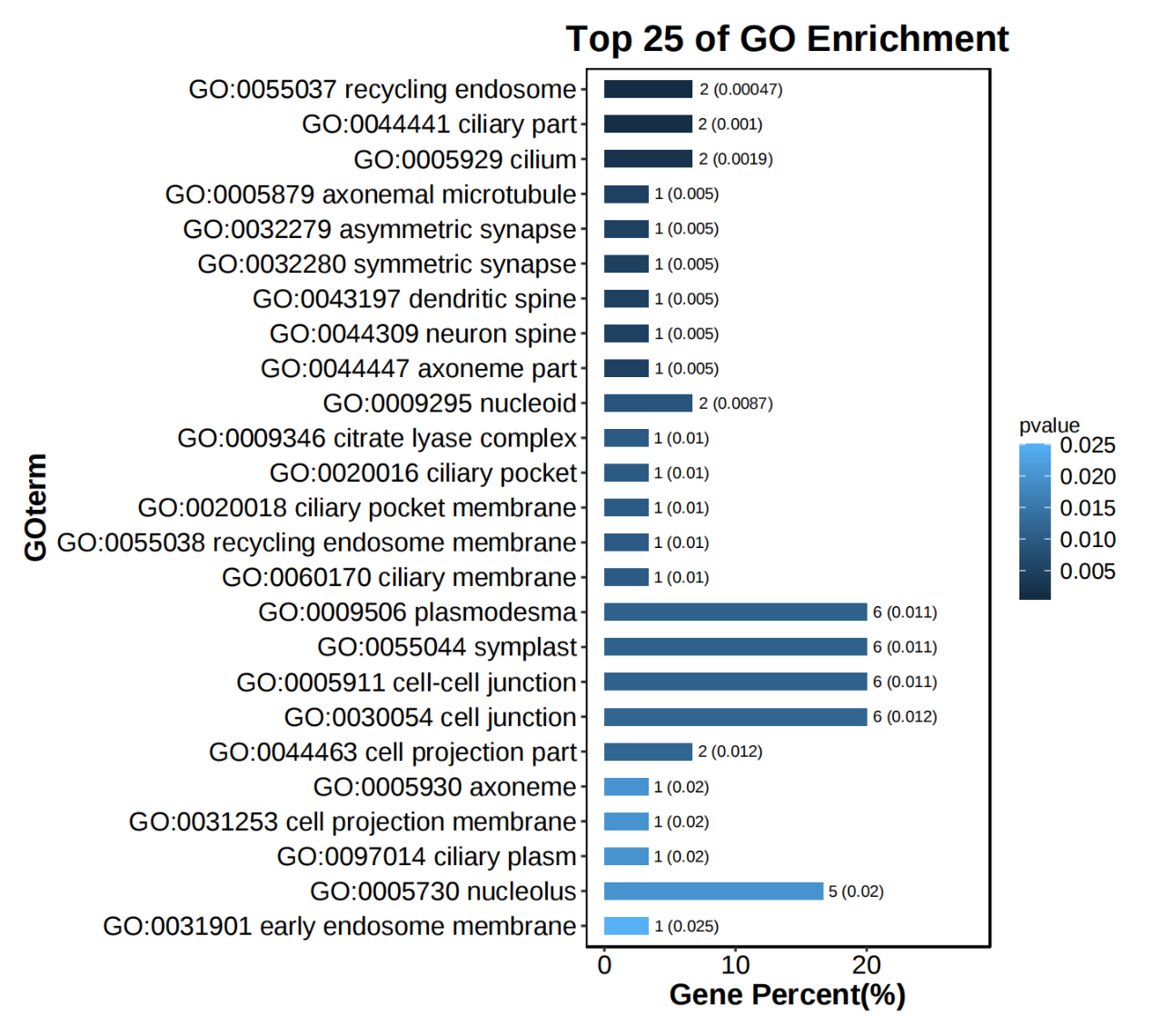
**

**Figure S7. Top 25 GO enrichment terms of the specially upregulated DEGs in the Ac5 root tuber.**

**
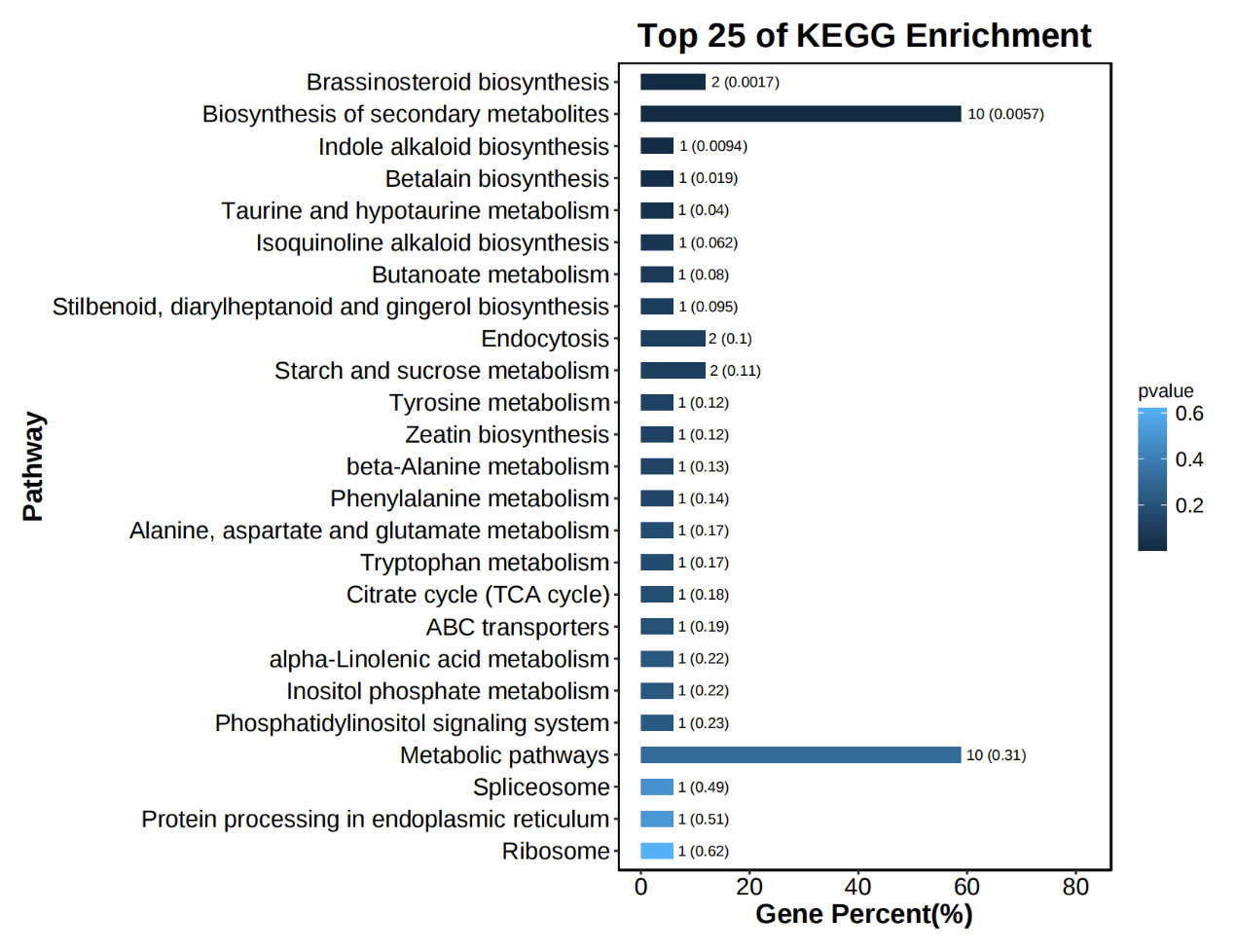
**

**Figure S8. Top 25 pathways in the KEGG enrichment analysis of the specially upregulated DEGs in the Ac5 root tuber.**
